# *Arabidopsis* GH3.10 conjugates jasmonates

**DOI:** 10.1111/plb.70001

**Published:** 2025-03-17

**Authors:** B. Ni, M. Klein, B. Hossbach, K. Feussner, E. Hornung, C. Herrfurth, M. Hamberg, I. Feussner

**Affiliations:** ^1^ Department of Plant Biochemistry, Albrecht‐von‐Haller‐Institute University of Goettingen Goettingen Germany; ^2^ Service Unit for Metabolomics and Lipidomics, Goettingen Center for Molecular Biosciences (GZMB) University of Goettingen Goettingen Germany; ^3^ Division of Physiological Chemistry II, Department of Medical Biochemistry and Biophysics Karolinska Institutet Stockholm Sweden; ^4^ Department of Plant Biochemistry, Goettingen Center for Molecular Biosciences (GZMB) University of Goettingen Goettingen Germany

**Keywords:** GH3 enzymes, jasmonate biosynthesis, jasmonate catabolism, oxylipins, phytohormones, seed formation, wound response

## Abstract

- Jasmonates regulate plant development and defence. In angiosperms, the canonical bioactive jasmonate is jasmonoyl‐isoleucine (JA‐Ile), which is formed in *Arabidopsis thaliana* by JAR1 and GH3.10. In contrast to other jasmonate biosynthesis or perception mutants, however, *gh3.10 jar1* knockout lines are still fertile. Therefore we investigated whether further jasmonates and GH3 enzymes contribute to regulation of fertility.
- Jasmonate levels were analysed by liquid chromatography–mass spectrometry. The substrate range of recombinant GH3.10 and related GH3 enzymes was studied using non‐targeted *ex vivo* metabolomics with flower and leaf extracts of *A. thaliana* and *in vitro* enzyme assays. Jasmonate application experiments were performed to study their potential bioactivity.
- In flowers and wounded leaves of *gh3.10 jar1* knockout lines JA‐Ile was below the detection limit. While 12‐hydroxy‐JA was identified as the preferred substrate of GH3.10, no other recombinant GH3 enzymes tested were capable of JA‐Ile formation. Additional JA conjugates found in wounded leaves (JA‐Gln) or formed in flowers upon MeJA treatment in the absence of JA‐Ile (JA‐Gln, JA‐Asn, JA‐Glu) were identified. The *aos gh3.10 jar1* was introduced as a novel tool to test for the bioactivity of JA‐Gln to regulate fertility.
- This study found JAR1 and GH3.10 are the only contributors to JA‐Ile biosynthesis in *Arabidopsis* and identified a number of JA conjugates as potential bioactive jasmonates acting in the absence of JA‐Ile. However, their contribution in regulating fertility is yet to be conclusively determined.

## Graphical abstract

12‐hydroxy jasmonate is directly converted into 12‐hydroxy jasmonoyl‐isoleucine and fertile jasmonoyl‐isoleucine deficient *Arabidopsis* mutants point towards the presence of other bioactive jasmonates in flowers.

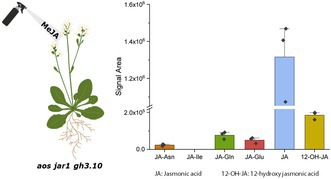

## INTRODUCTION

Plants employ highly sophisticated signalling networks to regulate their development or react to changes in the environment. Frequently, this requires a balance to be established between at times competing resource‐demanding processes, such as growth and defence (Huot *et al*. 2014; Zhou *et al*. 2022). At the signalling level, this is driven by a multitude of phytohormones that each regulate distinct downstream responses. In addition, cross‐talk between phytohormones in the form of mutual inhibition or response amplification contributes to the integration of developmental and environmental cues, thus orchestrating an appropriate plant response (Benavente & Alonso 2006; Sun *et al*. 2006; Chandler 2009; Van der Does *et al*. 2013; Zhang *et al*. 2016; Huang *et al*. 2023). An additional element of complexity is the possible rapid interconversion of many phytohormones between their bioactive and inactive forms, e.g., through their conjugation to amino acids. In the case of auxin (indole‐3‐acetic acid: IAA), the conjugation of IAA to amino acids marks IAA‐Ala for storage (Kowalczyk & Sandberg 2001), and IAA‐Asp and IAA‐Glu for degradation (Östin *et al*. 1998). The biosynthesis of such conjugates in plants is catalysed by a group of acyl adenylases, referred to as GRETCHEN HAGEN 3 (GH3) enzymes (Staswick *et al*. 2002). Phylogenetic analyses may divide all GH3 enzymes in *Arabidopsis thaliana* into three distinct clades based on sequence similarity and synteny (Okrent & Wildermuth 2011). To date, the 19 GH3 enzymes from *A. thaliana* have been studied to various degrees and the involvement of some of these enzymes in crucial biosynthetic pathways has been reported.

For instance, GH3.12/PBS3, a clade III member, was identified as a contributor to salicylic acid (SA) synthesis by catalysing the conjugation of the SA precursor isochorismate to glutamate, which subsequently decays spontaneously or is accelerated by EPS1 into SA (Rekhter *et al*. 2019; Torrens‐Spence *et al*. 2019). Many higher‐order mutants of IAA‐conjugating clade II GH3 enzymes exhibit reduced fertility, impaired root growth, or increased sensitivity to auxins (Casanova‐Sáez *et al*. 2022; Guo *et al*. 2022). Notably, jasmonic acid (JA) requires a GH3‐dependent activation through conjugation to an amino acid (Staswick & Tiryaki 2004). Jasmonates regulate a diverse array of developmental and defence processes and are defined as derivatives of JA (Wasternack & Hause 2002; Wasternack & Feussner 2018). In *Arabidopsis* and many other seed plants, jasmonate signalling is mediated through a receptor called CORONATINE INSENSITIVE 1 (COI1) (Devoto *et al*. 2002). COI1‐dependent signalling, often referred to under the umbrella term ‘jasmonate signalling’, is crucial for fertility in *Arabidopsis* (Cai *et al*. 2014). This is demonstrated by male sterility and failure to form mature siliques in both jasmonate biosynthesis and perception mutants, such as *aos* or *coi1* (Feys *et al*. 1994; von Malek *et al*. 2002). The isoleucine conjugate JA‐Ile is the principal bioactive COI1 ligand in most angiosperms. In *Arabidopsis*, GH3.11/JAR1 is the predominant enzyme for JA‐Ile production, but *jar1* plants still contain residual JA‐Ile (Suza & Staswick 2008). The other *Arabidopsis* GH3 clade I member, GH3.10, was only recently biochemically characterized as another contributor to JA‐Ile synthesis (Delfin *et al*. 2022). The loss of both GH3.10 and JAR1 causes severely reduced fertility in *Arabidopsis*, however *gh3.10 jar1* plants are still not sterile, suggesting the presence of yet another JA‐Ile producing enzyme or another jasmonate as COI1 ligand.

We report a distinct substrate preference of *Arabidopsis* GH3.10 for 12‐OH‐JA in addition to the properties it shares with JAR1. Furthermore, we present first evidence for additional bioactive jasmonates as facilitators of the preserved fertility in *gh3.10 jar1* plants. We introduce the *aos gh3.10 jar1* mutant as a new tool to study jasmonate signalling in the absence of JA‐Ile and other endogeneous jasmonates.

## MATERIAL AND METHODS

### Plant material and growth conditions

*Arabidopsis thaliana* wild‐type plant lines of the ecotype Col‐0 were used. In addition, mutant lines *jar1*‐1 (Staswick *et al*. 2002), *jar1*‐11 (SALK_034543), *jar1*‐12 (SALK_059774), *gh3.10* (SALKseq_036961.3), *coi1‐*1 (Benedetti *et al*. 1995), and *aos* (SALK_017756) were used. Seeds were stratified at 4°C for 2 days. For wounding experiments, plants were grown under short‐day conditions for 6 weeks (8 h/16 h light/dark cycle, 22°C, 60% humidity, and light intensity of 100 μmol m^−2^ s^−1^) in a plant growth cabinet (Percival CU‐36 L/D, Percival Scientific, Perry, USA). For all other experiments, plants were grown under long‐day conditions in a climate chamber (16 h/8 h light/dark cycle, 22°C, 60% humidity, and light intensity of 100 μmol m^−2^ s^−1^). Plants were wounded three times across the midvein using forceps and harvested 2 h later according to Mosblech *et al*. (2008). Flower material was harvested from flower stages 13 to 15 and pooled (Smyth *et al*. 1990). For *ex vivo* and metabolite fingerprinting studies of flowers and leaves, pooled material from *gh3.10 jar1‐*11 and *gh3.10 jar1*‐12 lines was used. For root growth assays, seedlings were grown in upright square Petri dishes on ½ Murashige Skoog agar with 50 μM MeJA. Seeds were sterilized in chlorine gas by incubation in a desiccator for 1 h after adding 50 mL 12% NaClO and 5 mL 32% HCl to a container at the bottom of the desiccator, below the seeds, and subsequently sown in a line across the top quarter of the Petri dish. Every Petri dish was photographed after 2 weeks and root length measured using Fiji (Schindelin *et al*. 2012).

### RNA extraction, cDNA synthesis, and transcriptomic analysis

The RNA was extracted from 100 mg homogenized plant material according to the manufacturer's instructions unsing the *Quick*‐RNA Plant Kit (Zymo Research Europe, Freiburg, Germany). RNA was sequenced and analysed at Novogene (UK). Samples were checked for quality via a Bionanalyzer (Agilent Technologies, Santa Clara, USA), and library preparation was performed based on polyA enrichment using directional mRNA library preparation. The libraries were sequenced using the NovaSeq 6000 platform (Illumina) with Novogene dual adaptors: 5′‐ AGATCGGAAGAGCGTCGTGTAGGGAAA GAGTGTAGATCTCGGTGGTCGCCGTATCATT‐3′ for read 1 and 5′‐GATCGGAAGAGCACACGTCTGAACTCCAGTCACGGATGACTATCTCGTATGCCGTCTTCTGCTTG‐3′.

For the cloning expression plasmids, RNA was extracted from 100 mg homogenized plant material using a phenol‐based protocol (Onate‐Sanchez & Vicente‐Carbajosa 2008). cDNA was generated using the RevertAid H Minus First Strand cDNA Synthesis Kit (Thermo Fisher Scientific).

### Protein expression and purification

The protein coding sequence of GH3.10 (At4g03400) was cloned into pET28a (Merck KGaA) from *Arabidopsis* cDNA using primers listed in Table S1. The pET28 expression plasmid containing JAR1 was kindly provided by Dr. Erich Kombrink, Max Planck Institute for Plant Breeding Research, Cologne, Germany. The pET28 plasmids containing GH3.8 (At5g51470), GH3.13 (At5g13350), GH3.14 (At5g13360), GH3.16 (At5g13380), GH3.18 (At1g48670), and GH3.19 (At1g48660) were generated from cDNA using primers listed in Table S1. pET28 expression plasmids for GH3.15 (At5g13370) and GH3.17 (At1g28130) were obtained in their codon‐optimized version from BioCat. Plasmids were transformed into *Escherichia coli* BL 21 Star (DE3) cells (Thermo Fisher Scientific) by heat shock (45 s at 42°C, followed by 5 min incubation on ice). Expression was induced in auto‐induction medium by incubating cell cultures at 37°C for 2 h and subsequently transferring them to 16°C for 3 days to obtain sufficient amounts of bacterial culture (Studier 2005). Cell pellets from expression cultures were resuspended in lysis buffer (20 mM Tris–HCl, pH 7.5, 20 mM imidazole, 1 mM DTT, 500 mM NaCl, 1 mg mL^−1^ Lysozyme, 0.1 mg mL^−1^ DNAseI, 0.1 mM PMSF) and disrupted using an ultrasonic tip. Lysate was cleared by centrifugation at 50,000×*g* for 45 min at 4°C. The supernatant was loaded onto a HisTrap column (Cytiva) using the Äkta Prime system (Cytiva) and His‐tagged GH3 enzymes were eluted by applying an elution buffer (20 mM Tris–HCl, pH 7.5, 500 mM imidazole, 1 mM DTT, 500 mM NaCl) gradient (elution at 25–30% elution buffer). Eluted protein was collected and concentrated using centrifugal concentrators with a 30 kDa molecular weight cut‐off (Corning, NY, USA) and the buffer was gradually changed to GH3 storage buffer during the process (10 mM Tris–HCl, pH 7.5, 1 mM DTT, 1 mM MgCl_2_, 20 mM NaCl). Enzymes were either used directly after purification or snap‐frozen in liquid nitrogen and stored at −80°C.

### Metabolite extraction

Metabolites from flower and leaf tissues were extracted based on a workflow modified from Matyash *et al*. (2008) and Feussner *et al*. (2023). For *ex vivo* studies and metabolite fingerprinting, 100 mg homogenized flower or leaf material were extracted according to a method described in Feussner & Feussner (2019). Homogenized material was mixed with 750 μL methanol and 2.5 mL methyl *tert*‐butyl ether (MTBE) and shaken in the dark at 4°C for 1 h. After addition of 600 μL H_2_O and the resulting phase separation, the upper phase was transferred to a new glass vial and the lower phase was re‐extracted by addition of a 700 μL methanol/water (3.0:2.5 v/v) mix and 1.2 mL MTBE, after which all upper phases (from first phase separation and after re‐extraction) and the lower phase were combined and dried under a stream of nitrogen. We used this procedure primarily to remove this cell debris, since lighter plant tissue particles (especially parts of the petals) tended to accumulate at the interphase. Therefore we later combined both phases again since we wanted to analyse as many metabolites as possible over the whole range of polarities. Dried extracts were resuspended in 200 μL methanol and 100 μL water and stored at −20°C. For *ex vivo* assays, 100 μL of the extract were dried under a stream of nitrogen and re‐dissolved in 100 μL *ex vivo* assay buffer.

### Jasmonate quantification

The jasmonate content of flowers and leaves 2 h post‐wounding (hpw) of *Arabidopsis* plant lines was analysed by ultra high pressure liquid chromatography‐nano‐electrospray ionization coupled to tandem mass spectrometry (UHPLC‐nanoESI‐MS/MS) as described in Herrfurth & Feussner (2020). Metabolites were reverse‐phase separated on an ACQUITY UPLC HSS T3 column (100 × 1 mm, 1.8 μm particle size; Waters) via an ACQUITY UPLC system (Waters) and analysed by chip nanoESI (TriVersa Nanomate; Advion BioSciences) coupled with an AB Sciex 4000 QTRAP tandem mass spectrometer (AB Sciex) in scheduled multiple reaction monitoring mode, with the following mass transitions: 209/59 (declustering potential (DP) −30 V, entrance potential (EP) −4.5 V, collision energy (CE) −24 V) for JA, 214/62 (DP −35 V, EP −8.5 V, CE −24 V) for D5‐JA, 225/59 (DP −35 V, EP −9 V, CE −28 V) for 12‐OH‐JA, 291/165 (DP −50 V, EP −5 V, CE −26 V) for OPDA, 296/170 (DP −65 V, EP −4 V, CE −28 V) for D5‐OPDA, 308/116 (DP −45 V, EP −5 V, CE −28 V) for JA‐Val, 322/130 (DP −45 V, EP −5 V, CE −28 V) for JA‐Ile, 325/133 (DP −65 V, EP −4 V, CE −30 V) for D3‐JA‐Leu, 337/145 (DP −45 V, EP −5 V, CE −28 V) for JA‐Gln, and 338/130 (DP −45 V, EP −10 V, CE −30 V) for 12‐OH‐JA‐Ile.

### The GH3 *in vitro* activity assay

The ability of 10 *Arabidopsis* GH3 enzymes (GH3.8, GH3.10, GH3.11/JAR1, GH3.13, GH3.14, GH3.16, GH3.17, GH3.18, GH3.19) to form JA‐Ile and 12‐OH‐JA‐Ile was tested by *in vitro* incubations with JA or 12‐OH‐JA, ATP and Ile. 100 μg, purified enzyme were incubated in 100 μL reactions mix (10 mM Tris–HCl, pH 7.5, 1 mM ATP (stock adjusted to pH 7.5 with NaOH), 5 mM MgCl_2_, 10% (v/v) glycerol, 1 mM DTT, 0.5 mM Ile and 0.1 mM JA or 12‐OH‐JA) for 1 h at 30°C while shaking gently. Reactions were stopped by addition of 50 μL LC–MS‐grade acetonitrile, and samples were centrifuged at 20,800×*g* to remove precipitated protein before UHPLC‐HRMS analysis.

### Determination of GH3.10 and JAR1 relative activity

The crystal structure of JAR1 with JA‐Ile (PDB ID 4EPL; Westfall *et al*. 2012) was used for the structural modelling of GH3.10 with 12‐OH‐JA‐Ile. The structure prediction of GH3.10 was obtained from the Robetta structure prediction service (Baek *et al*. 2021). GH3.10 was superimposed on the structure of JAR1 and the co‐crystalized ligand JA‐Ile was swapped for 12‐OH‐JA‐Ile. For the determination of relative enzyme activity, GH3.10 and JAR1 were purified in parallel and tested for enzyme activity by *in vitro* incubations with 4,5‐didehydro JA (4,5‐ddh‐JA), JA, or 12‐OH‐JA with ATP and Ile. 100 μg purified enzyme were incubated in 100 μL reaction mix (10 mM Tris‐HC, l pH 7.5), 1 mM ATP (stock adjusted to pH 7.5 with NaOH), 5 mM MgCl_2_, 10% (v/v) glycerol, 1 mM DTT, 1 mM Ile, and 0.1 mM JA, 12‐OH‐JA or 4,5‐ddh‐JA for 1 h at 30°C while shaking gently. JA was obtained from Merck KGaA (Darmstadt, Germany), 12‐OH‐JA was obtained from Toronto Research Chemicals (Toronto, Canada), and 4,5‐didehydro JA was kindly provided by Mats Hamberg, Karolinska Institutet, Stockholm, Sweden. Reactions were stopped by addition of 50 μL acetonitrile LC–MS‐grade and analysed by ultra‐high‐performance liquid chromatography‐high resolution mass spectrometry (UHPLC‐HRMS).

### GH3.10 *ex vivo* analysis

For e*x vivo* reactions, 100 μL plant metabolite extract were dried under a nitrogen stream and resuspended in 100 μL GH3 *ex vivo* assay buffer (20 mM Tris–HCl, pH 7.5, 1 mM ATP (stock adjusted to pH 7.5 with NaOH), 5 mM MgCl_2_, 1 mM DTT, 1 mM Ala, Arg, Asn, Asp, Cys, Glu, Gln, Gly, His, Ile, Leu, Lys, Met, Phe, Pro, Ser, Thr, Trp, Tyr, Val). Samples were sonicated for 10 min at room temperature, and 100 μg purified GH3.10 added to each sample. For inactive enzyme control samples, half a volume of acetonitrile LC–MS‐grade was added to enzyme stocks. *Ex vivo* reactions were incubated for 1 h at 30°C while shaking gently, and reactions were stopped by addition of 50 μL acetonitrile LC–MS‐grade. Samples were centrifuged at 20,800×*g* to remove precipitated protein before UHPLC‐HRMS analysis.

### Generation of JA‐Gln and JA‐Ile and jasmonate plant treatment experiments

Jasmonate conjugates were generated by incubation of JA, ATP and Gln or Ile with JAR1 and GH3.15, respectively, as described previously (Sherp *et al*. 2018). 20 mg JA were added to 100 mL reaction mix (20 mM Tris–HCl, pH 7.5, 2 mM ATP (stock adjusted to pH 7.5 with NaOH), 5 mM MgCl_2_, 1 mM DTT, 2 mM Ile or Gln) and incubated at room temperature overnight with 40–60 mg JAR1 (for JA‐Ile production) or GH3.15 (JA‐Gln production). Subsequently, 50 mL acetonitrile LC–MS‐grade were added. Precipitated protein was removed through centrifugation (15 min at 3220×*g*, 4°C) and the supernatant checked for substrate‐turnover by UPLC‐HRMS. Under our conditions, more than 99% of the added JA was metabolized to the respective conjugates. The supernatant was completely dried using a rotation evaporator (Rotavapor® R‐220; BÜCHI Labortechnik). Jasmonates were redissolved and separated from precipitated buffer components by sequentially adding two times 20 mL methanol LC–MS‐grade. The solutions were dried under a nitrogen stream and respuspended in water as 10 mM stock solutions. For jasmonate treatment experiments, MeJA, JA, JA‐Gln, and JA‐Ile were diluted in water containing 0.1% Tween‐20 to a final concentration of 500 μM or 440 μM in the case of MeJA. Six‐ to 10‐week‐old *Arabidopsis* plants (Col‐0, *aos* and equal amounts of *aos gh3.10 jar1*‐11 and *aos gh3.10 jar1*‐12) were sprayed with the jasmonates daily until dripping wet (20 mL for 12 plants) over the course of 2 to 4 weeks. Water with 0.1% Tween‐20 served as mock treatment.

## RESULTS

In *Arabidopsis*, JAR1 has been characterized as the main JA‐Ile‐forming enzyme. However, residual levels of JA‐Ile remain in *jar1* plants, which result in fertile plants (Suza & Staswick 2008; Staswick 2009). This indicates the existence of additional JA conjugating enzymes. Recently, GH3.10 was identified as second JA‐Ile conjugating enzyme in *Arabidopsis* (Delfin *et al*. 2022). Therefore we investigated its role and the presence of yet another JA‐Ile‐producing enzyme.

### The *gh3.10 jar1* plants show reduced silique formation and loss of jasmonate‐induced root growth inhibition

**Fig. 1:**
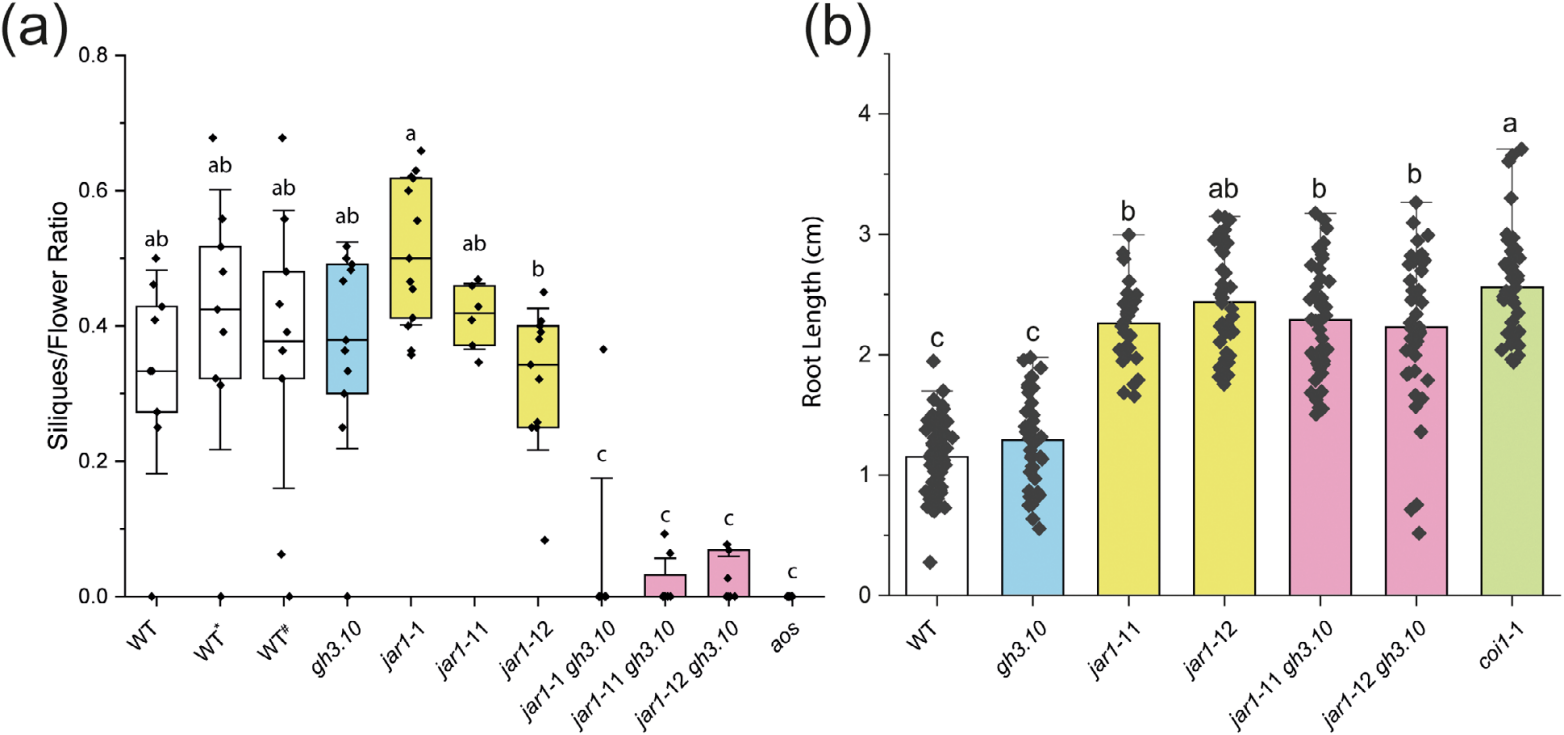
*gh3.10 jar1* plants exhibit significantly reduced silique formation but no increased sensitivity to MeJA in roots. (a) The silique maturation rate of 6‐week‐old plants was determined as the ratio of siliques to overall flowers per plant. Col‐0 plants served as wild‐type control. * and # indicate wild‐type control lines backcrossed from *jar1*‐11 and *jar1*‐12, respectively. (b) Root growth of *gh3.10 jar1* plants was assessed by growing seedlings on ½ MS agar containing 50 μM MeJA. Statistical analysis was performed by one‐way ANOVA with Tukey's *post‐hoc* test. Mean ± SD of three technical replicates are shown. Letters denote significant differences at *p* < 0.05. aos: allene oxide synthase, coi1: coronatine insensitive 1.

Since male fertility is not abolished in *jar1* plants, fertility was assessed in *gh3.10 jar1* double knockout lines by determining the ratio of siliques to overall flowers. In wild‐type, *gh3.10* and *jar1* lines, ca. 30%–60% of all flowers had developed into siliques (Fig. 1a). Silique formation rate of *gh3.10 jar1* plants, however, was significantly reduced to <10, % along with significantly delayed silique maturation, suggesting residual jasmonate signalling.

Methyl jasmonate (MeJA) inhibits growth of the primary root in wild‐type plants, which is partly lost in *jar1* plants (Staswick *et al*. 2002). The effect of the loss of both JAR1 and GH3.10 was investigated in *Arabidopsis* seedlings grown on ½ MS agar containing 50 μM MeJA. Presence of MeJA led to a slight, but not significant, increase of root length in *gh3.10* plants (Fig. 1b). The loss of *jar1* caused a significant increase in root length to twice the length of the wild‐type Col‐0 seedlings. However, in *gh3.10 jar1* there was no additional increase in root length beyond that observed for *jar1* and *coi1*‐1. This suggested that JAR1 is the key contributor to jasmonate signalling in roots.

### Loss of GH3.10 in addition to JAR1 abolishes JA‐Ile formation in *gh3.10 jar1* plants

**Fig. 2:**
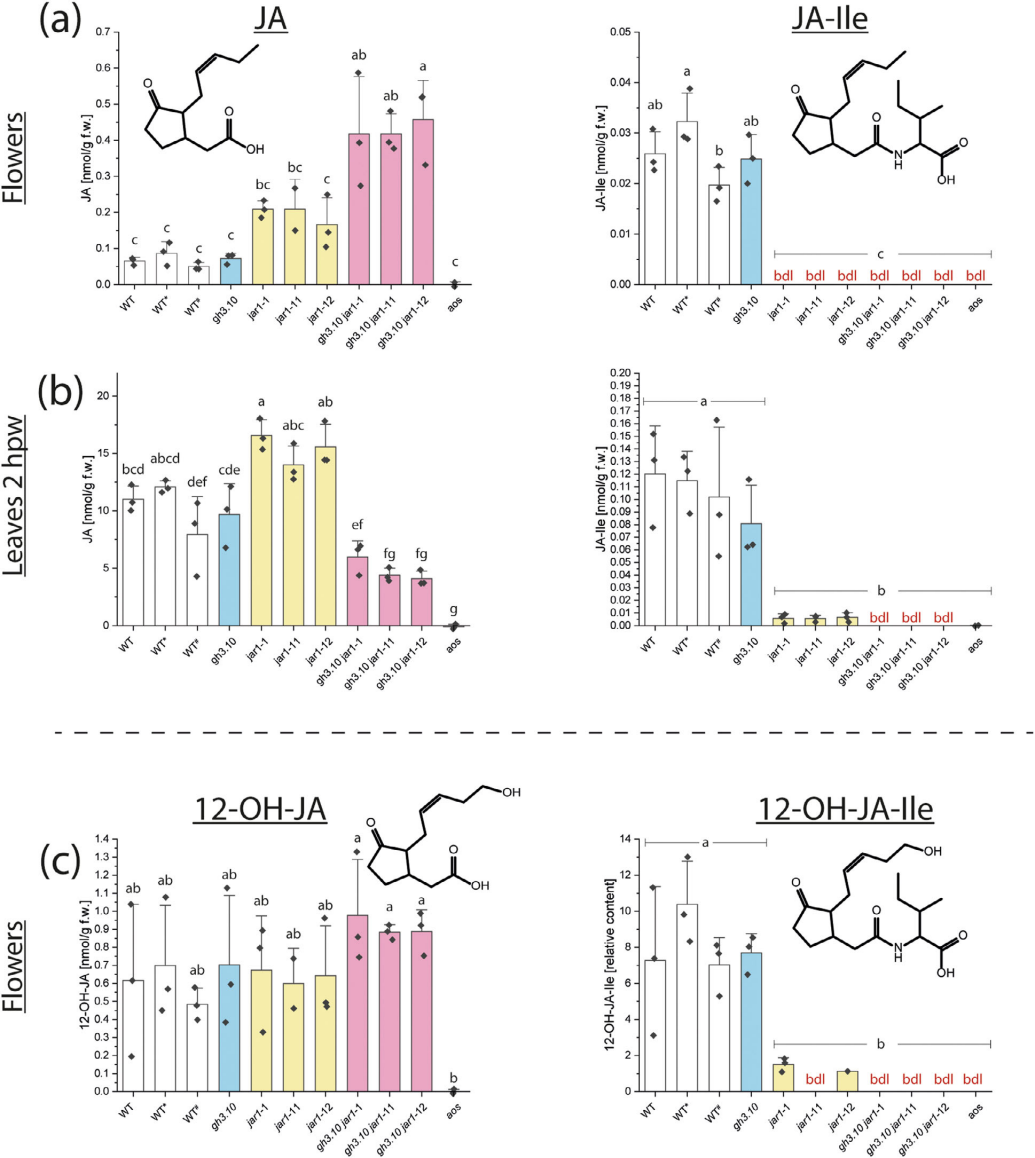
Jasmonate levels in flowers and wounded leaves of *gh3.10 jar1* plants. JA and JA‐Ile levels were determined in flowers (a) and leaves at 2 hpw (b) of *gh3.10 jar1* plant lines. Col‐0 plants served as wild‐type control. * and # indicate wild‐type control lines backcrossed from *jar1*‐11 and *jar1*‐12, respectively. Levels of 12‐OH‐JA and 12‐OH‐JA‐Ile (c) were measured in flowers. Amounts of JA‐Val and JA‐Ile in flowers and wounded leaves are shown in Fig. S4. Statistical analysis was performed by one‐way ANOVA with Tukey's *post‐hoc* test. Mean ± SD of three technical replicates are shown. Letters denote significant differences at *p* < 0.05. aos: allene oxide synthase, bdl: below detection limit.

Based on their reduced fertility, jasmonate levels in the flowers of *gh3.10 jar1* plants were determined (Fig. 2). Wounding is a major trigger of jasmonate‐ ormation and was additionally used to investigate accumulation of JA‐Ile (Glauser *et al*. 2009; Koo & Howe 2009). Therefore, we first performed a wounding time course for up to 6 h week^−1^ (hpw; Fig. S1). Since all jasmonates were detected at 2 hpw, this time point was further analysed. Jasmonate‐deficient *aos* plants served as negative control and measurements were noise‐corrected through data obtained from analysis of the jasmonate‐free *aos* samples. In flowers, *jar1* plants accumulated JA to approximately twice the level of wild‐type and *gh3.10* plants (Fig. 2a). JA levels of *gh3.10 jar1* increased fourfold compared to wild‐type and *gh3.10*. Since the differences in the constitutive jasmonate levels in leaves of wildtype and all mutant lines were not significant, wounded leaves were analyzed. In wounded leaves of *gh3.10 jar1*, JA was detected at 50% of wild‐type levels (Fig. 2b). Preliminary JA‐Ile quantification through highly sensitive MRM‐based LC–MS‐analyses in the flowers and wounded leaves of *gh3.10 jar1* plant lines also revealed residual signals similar to those of JA‐Ile. However, these weak signals were also detected in jasmonate‐free *aos* and an empty extraction control sample (Fig. S2). Therefore we regard these signals rather as background noise, rather than as specific signals. All jasmonate data were therefore noise‐corrected through the respective background *aos* sample signal, after which no JA‐Ile could be detected in either flowers or wounded leaves of *gh3.10 jar1* plants. In flowers, JA‐Ile was detected at the same level in both wild‐type and *gh3.10* plants and was below the detection limit of 0.017 nmol g^−1^ FW in flowers of *jar1* and *gh3.10 jar1* plants. In wounded leaves, JA‐Ile accumulated to similar levels in wild‐type and *gh3.10* plants. Residual JA‐Ile levels were detected in *jar1* plants, but in *gh3.10 jar1* plants JA‐Ile was below the detection limit (0.00085 nmol g^−1^ FW in leaf samples). In addition to JA‐Ile, the levels of JA‐Val and JA‐Leu were measured in wounded leaves and flowers (Fig. S3) as well as determination of 12‐OH‐JA and 12‐OH‐JA‐Ile in flowers (Fig. 2c). Again, JA‐Val, JA‐Leu and 12‐OH‐JA‐Ile were detected at reduced levels in *jar1* plants and were below the detection limit in *gh3.10 jar1* lines, while 12‐OH‐JA increased. Together these results strongly suggest that the loss of both GH3.10 and JAR1 abolishes JA‐Ile, JA‐Leu, and JA‐Val formation, and that they are exclusively a product of JAR1 and GH3.10.

### Non‐targeted *ex vivo* metabolomics and targeted *in vitro* reactions reveal 12‐OH‐JA as substrate of GH3.10

**Fig. 3:**
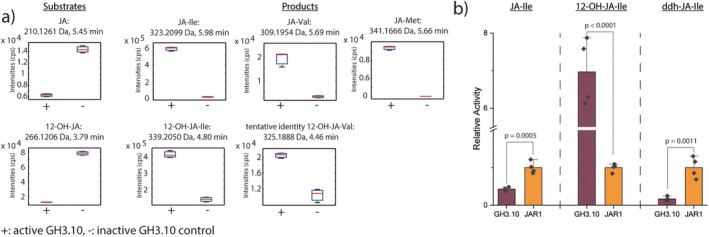
Non‐targeted *ex vivo* metabolomics and targeted *in vitro* studies reveal JA and 12‐OH‐JA as substrates for GH3.10. (a) For *ex vivo* studies, recombinant purified GH3.10 was incubated with total metabolite extract from Col‐0 leaves at 2 hpw. Samples were measured by UHPLC‐HRMS. Substrates and products of GH3.10 were identified by the relative intensitiy pattern of metabolite features in samples incubated with active enzyme compared to inactive controls. JA and 12‐OH‐JA were identified as substrate metabolites in addition to the respective products, JA‐Ile, JA‐Met, and JA‐Val, as well as 12‐OH‐JA‐Ile and tentative 12‐OH‐JA‐Val. Results shown are representative for similar results obtained from the *gh3.10 jar1* leaves 2 hpw dataset (Fig. S4). The identity of the metabolites was confirmed by UHPLC‐HRMSMS analyses, in‐source fragments, and reproduction of reactions *in vitro*. Data represent n = 3 replicates (pooled samples from five wounded plants). (b) The relative conversion rate of JA, 12‐OH‐JA, and ddh‐JA by JAR1 and GH3.10. Activity was tested by *in vitro* incubations with Ile and substrates 12‐OH‐JA, JA and ddh‐JA, and measured by UHPLC‐HRMS. 100 μg purified enzyme were incubated in reaction buffer containing 1 mM Ile and 0.1 mM JA, 12‐OH‐JA or ddh‐JA. Reactions were incubated for 1 h at 30°C while gently shaking and stopped by addition of acetonitrile. Signal area relative to respective mean JAR1 signal for each substrate is shown. Mean ± SD of n = 4 technical replicates are shown. Brackets indicate significant differences as determined by Student's *t*‐test at *p* < 0.05.

To obtain a comprehensive overview on GH3.10 substrate preference, non‐targeted *ex vivo* metabolomics was performed with recombinant GH3.10 (Ni & Feussner 2023). Metabolite extracts were isolated from Col‐0 leaves at 2 hpw, representing conditions showing high *GH3.10* expression, to provide a quasi‐native substrate environment for the enzyme. The metabolite extract was supplemented with ATP and 20 proteinogenic amino acids to ensure the availability of GH3 co‐substrates. Inactivated enzyme served as control. Potential substrates and products of the GH3.10 *ex vivo* reaction were predicted from the relative intensities detected in active enzyme samples compared to inactive controls, as well as amino acid‐based mass shifts. Tentative metabolite identities were confirmed by targeted MS/MS analysis, in‐source fragments, retention time, and replications of reactions *in vitro*. *Ex vivo* assays with active GH3.10 showed reduced JA levels in leaf extract of wounded of Col‐0 plants, while the amount of JA‐Ile increased in samples incubated with active GH3.10 (Fig. 3). This pattern is characteristic for a substrate–product pair. In addition to JA‐Ile, JA‐Met and JA‐Val were identified as further jasmonoyl‐amido conjugates generated by GH3.10, similar to earlier reports on JAR1 activity (Staswick *et al*. 2002). 12‐OH‐JA was identified as a novel substrate of GH3.10, with its product 12‐OH‐JA‐Ile. This revealed the previously undescribed synthesis of 12‐OH‐JA‐Ile. The *ex vivo* assay was performed with three additional metabolite extracts as a control and to test the broadest substrate range possible. Wounded leaf material from *gh3.10 jar1* plants was included to account for the potential enrichment of GH3.10 substrates in the absence of endogenous JAR1 and GH3.10 (Fig. S4). The identified substrate–product pairs were in complete agreement with previous results. To cover an additional part of the metabolome for the GH3.10 *ex vivo* analysis, flower material of wild‐type and *gh3.10 jar1* plants was used. Here only JA‐Ile was confirmed as product generated by GH3.10.

Since both JA and 12‐OH‐JA were identified as substrates for GH3.10 through *ex vivo* metabolomics, substrate preference was compared to that of JAR1 using an *in vitro* enzymatic assay and UHPLC‐HRMS analysis. 4,5‐didehydro jasmonic acid (4,5‐ddh‐JA) was included in addition because of its structural similarity to JA and its reported presence in the cytosol (Chini *et al*. 2018). In comparison to JAR1, GH3.10 exhibited approximately half the relative activity towards JA. Both GH3.10 and JAR1 produced ddh‐JA‐Ile *in vitro*; however, JAR1 showed 6‐fold higher relative activity with 4,5‐didehydro‐JA compared to GH3.10 (Fig. 3b). JAR1 was also capable of conjugating 12‐OH‐JA to 12‐OH‐JA‐Ile; however, GH3.10 exhibited a notably higher 7‐fold relative activity towards 12‐OH‐JA, opening up the possibility of a physiologically similar, but distinct, role of GH3.10 over JAR1.

To determine possible structural determinants of substrate preference of GH3.10 compared to JAR1, GH3.10 active site mutations were generated. A crystal structure of JAR1 and the predicted crystal structure of GH3.10 were used to compare the active sites (Fig. S5) (Westfall *et al*. 2012; Jumper *et al*. 2021; Baltzis *et al*. 2022). In the case of JAR1, the 3‐keto group of the jasmonate moiety is coordinated by histidine 328 (H^328^) via a water molecule. Notably, this histidine is replaced by alanine (A^334^) in GH3.10. To assess the effect of H^328^ in GH3.10, A^334^ was replaced by histidine. Secondly, tyrosine 172 (Y^172^) was replaced with valine due to its predicted close proximity to the hydroxy terminus of 12‐OH‐JA‐Ile. In addition, a GH3.10 variant with both active site mutations was generated. Neither of the mutations, alone or in combination, shifted the relativity of GH3.10 for jasmonate substrates (Fig. S6). This suggests that other structural determinants cause the prefererence of GH3.10 for 12‐OH‐JA compared to JAR1.

### The formation of JA‐Ile among *Arabidopsis* GH3 enzymes is exclusive to GH3.10 and JAR1 in *in vitro* studies

**Fig. 4:**
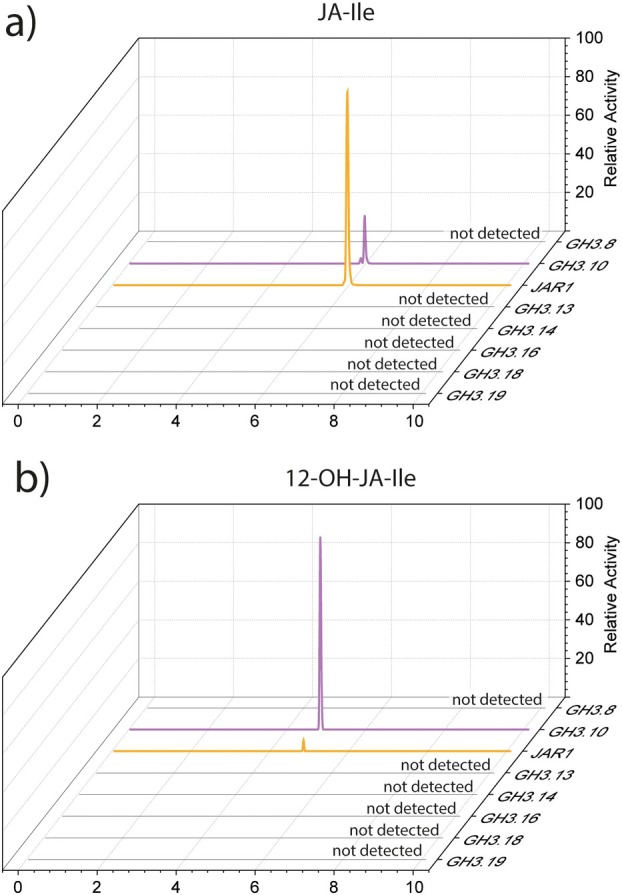
Investigation of further *Arabidopsis* GH3 enzymes contributing to JA‐Ile or 12‐OH‐JA‐Ile synthesis. 100 μg purified GH3 enzymes (GH3.8, GH3.10, GH3.11/JAR1, GH3.13, GH3.14, GH3.16, GH3.17, GH3.18, GH3.19) were incubated for 1 h at 30°C in reaction buffer containing 0.5 mM Ile and 0.1 mM JA or 12‐OH‐JA. Reactions were stopped by addition of acetonitrile, and (a) JA‐Ile and (b) 12‐OH‐JA‐Ile formation checked by UHPLC‐HRMS. Chromatograms shown indicate the relative JA‐Ile or 12‐OH‐JA‐Ile product signals generated by GH3.10 and JAR1.

The ability of further *Arabidopsis* GH3 enzymes to catalyse this conjugation was investigated. To date, clade II GH3 enzymes are well‐described as auxin‐conjugating enzymes and were consequently excluded from the experiment (Park *et al*. 2007; Gutierrez *et al*. 2012; Ostrowski *et al*. 2014; Aoi *et al*. 2019; Di *et al*. 2021; Guo *et al*. 2022). Instead, the focus was shifted to the less‐studied clade III GH3 enzymes. Here, the functions of GH3.12/PBS3, GH3.7 and GH3.15 so far have been mainly investigated *in vitro* (Sherp *et al*. 2018; Holland *et al*. 2019; Rekhter *et al*. 2019; Brunoni *et al*. 2023). Based on this selection, a comprehensive *in vitro* enzyme assay was performed with GH3.10, JAR1, GH3.8, GH3.13, GH3.14, GH3.16, GH3.18, and GH3.19. All enzymes were expressed in *E. coli*, purified and incubated at concentrations of 2 mg mL^−1^ in reaction mixes containing 1 mM JA, 1 mM Ile and 1 mM ATP for 1 h at 30°C. JA‐Ile formation was subsequently analysed by UHPLC‐HRMS. In separate control experiments, the activity of all clade III enzymes was confirmed by *ex vivo* metabolomics assays or *in vitro* reactions with a variety of acyl substrates. Among all enzymes tested, only JAR1 and GH3.10 were capable of forming JA‐Ile and 12‐OH‐JA‐Ile, thus pointing towards a unique function, being exclusive to the two clade I enzymes (Fig. 4).

### Transcriptomic analysis did not identify further GH3 enzymes with increased transcription rate in flowers of *gh3.10 jar1* plants

**Fig. 5:**
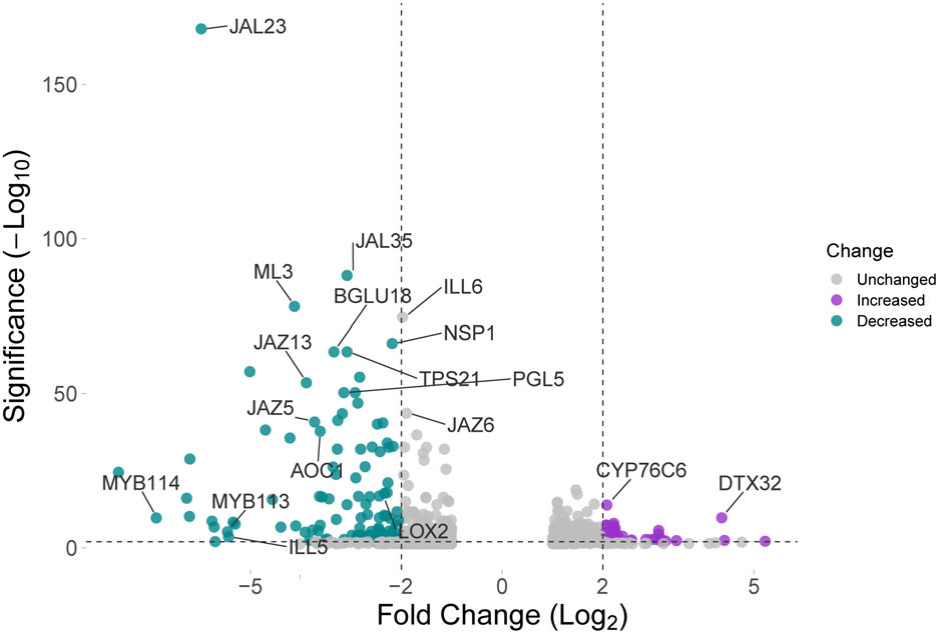
Volcano plot of differentially expressed genes of *gh3.10 jar1* and Col‐0 flowers. Each gene is represented by one dot.The log_2_ fold change indicates the mean expression level for each gene. Grey dots represent genes with no significantly different expression between *gh3.10 jar1* and Col‐0 (p > 0.01). Purple dots represent 37 genes with significantly increased expression (log_2_ fold change ≥2, *p* < 0.01) and cyan dots represent 127 genes with significantly reduced expression levels (log_2_ fold change ≤ −2, p < 0.01). Selected genes are indicated by name. Plot based on Goedhart & Luijsterburg (2020).

Both JAR1 and GH3.10 exert a profound influence over the phenotype of *Arabidopsis* plants. The loss of both enzymes prevents synthesis of JA‐Ile and thereby causes downstream effects due to the transcriptional control and regulatory functions of JA‐Ile (Pauwels *et al*. 2009; Hickman *et al*. 2017). To gain further insights into physiological changes resulting from the loss of JA‐Ile, the flower transcriptome of three independent *gh3.10 jar1* lines was compared to that of wild‐type Col‐0 plants. The aim was to identify other GH3 enzymes or other hormone‐derived signalling pathways that might be transcriptionally upregulated to compensate for the loss of GH3.10 and JAR1. In the mutant lines, the expression of 37 genes increased and 127 genes decreased (Log_2_ fold change ≥2 or ≤ −2 with *p* < 0.01). Neither a *GH3* transcript nor transcripts encoding for other hormone‐derived signalling pathways were among the genes identified here. Of the genes with the most strongly reduced expression levels, many were linked either directly to jasmonate metabolism (*LOX2, AOC1, ILL5*, and *ILL6*) or jasmonate signalling (*JAZ5*, *JAZ6, JAZ13, MYB113*, and *MYB114*; Fig. 5). In addition, a number of immunity‐related genes was also downregulated: *TPS21* (Terpene synthase 21), two jacalin‐related lectins *JAL23* and *JAL35*, and the β‐glucosidase *BGLU18*. Among the 37 genes with significantly increased expression, no clear association with jasmonate‐dependent regulation was identified. DTX32 belongs to the MATE (multidrug and toxic compound extrusion) family of efflux transporters, which facilitate the extrusion of toxic compounds from the cytoplasm (Li *et al*. 2002). Likewise, CYP76C6 belongs to a cytochrome P450 family that is involved in the metabolism of monoterpenols and herbicides, although the exact substrates of CYP76C6 are unknown (Höfer *et al*. 2014). These data support previous findings that JA‐Ile is a major regulator of the plant defence response and revealed no other GH3 enzyme or phytohormone pathway that may complement the loss of GH3.10 and JAR1 at the transcriptional level.

### The *aos gh3.10 jar1* triple mutant as a novel tool to study jasmonate‐derived fertility indicates that residual fertility of *gh3.10 jar1* plants is due to a JA‐derived signal

**Fig. 6:**
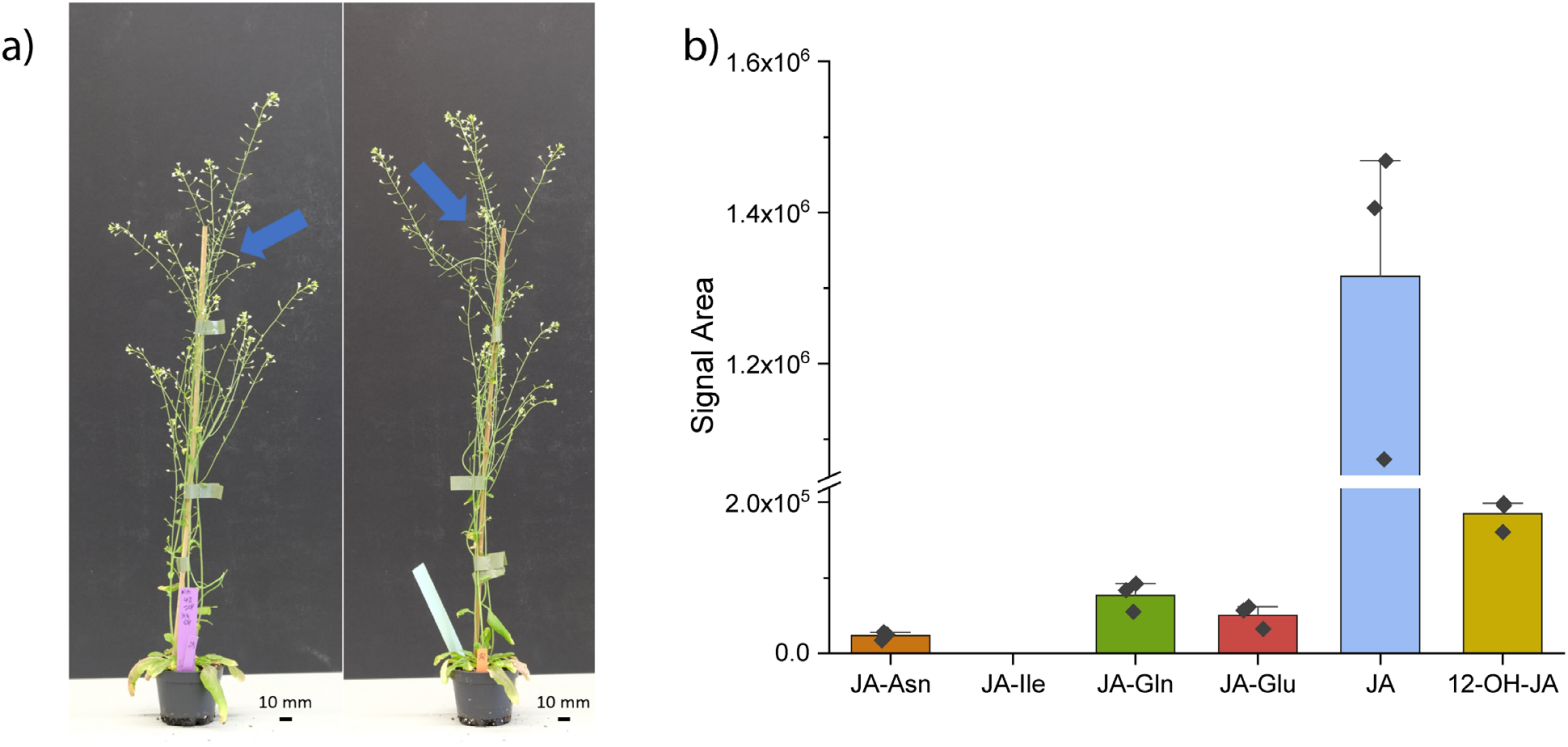
MeJA treatment of *aos gh3.10 jar1* plants partially restores fertility, and flowers show an enrichment of JA‐Gln, JA‐Glu, and JA‐Asn. Six‐week‐old *Arabidopsis aos gh3.10 jar1* plants were sprayed daily with buffer containing 440 μM MeJA and 0.1% Tween‐20 for 2 weeks. (a) The *aos*‐derived male sterility was partially rescued by the treatment, and plants produced siliques at reduced rates (siliques indicated by blue arrows). Flowers of stages 13–15 were harvested 2 h after the last MeJA treatment and analysed for jasmonate content using UHPLC‐HRMS. (b) Signal area of jasmonates and jasmonate conjugates in *aos gh3.10 jar1* flowers after MeJA treatment. Mean ± SD of three technical replicates are shown. bdl: below detection limit.

Both non‐targeted *ex vivo* metabolomics experiments and targeted *in vitro* reactions demonstrated the ability of GH3.10 and JAR1 to form the bioactive jasmonate JA‐Ile. Among the 10 tested *Arabidopsis* GH3 enzymes, this reaction was exclusive to GH3.10 and JAR1. However, *gh3.10 jar1* plants still show residual fertility despite the lack of detectable JA‐Ile. This suggests for the presence of a yet unidentified bioactive jasmonate or jasmonate precursor that functions as a ligand of COI1. In the liverwort *Marchantia polymorpha*, dinor*‐cis*‐12‐oxo‐phytodienoic acid (dn*‐cis*‐OPDA), *dinor‐iso*‐12‐oxo‐phytodienoic acid (dn*‐iso*‐OPDA), as well as Δ^4^‐dn‐*cis*‐OPDA and Δ^4^‐dn‐*iso*‐OPDA were identified as the ligand of MpCOI1, replacing the role of JA‐Ile in vascular plants (Monte *et al*. 2018; Kneeshaw *et al*. 2022). To test whether OPDA or other jasmonate precursors of JA are responsible for the remaining fertility observed in *gh3.10 jar1* plants by acting as COI1‐ligands, *aos gh3.10 jar1* mutant plants were generated. These mutant lines combine the inability to produce JA‐Ile with the absence of JA biosynthesis intermediates, thus abolishing possible endogenous jasmonate precursor‐derived signals (see reaction scheme, Fig. S7). Here, the aim was to test for a rescue of fertility after the application of candidate bioactive jasmonates. First, *aos gh3.10 jar1* flowers were sprayed with MeJA to check for a restoration of fertility. This was expected only for a bioactive signal downstream of JA. In the case of OPDA or dn‐OPDA acting as residual signal, the plants were expected to remain sterile as the signal‐activating isoleucine conjugation step is missing. Spraying MeJA over the course of 2 weeks restored fertility to 4 out of 16 MeJA‐treated *aos gh3.10 jar1* plants, which formed only 1 to 2 siliques per plant (see Fig. 6a). This suggested the presence of another JA‐Ile and 12‐OH‐JA‐Ile‐independent bioactive signal derived from JA (Fig. S7). The flowers of *aos gh3.10 jar1* plants treated with MeJA were harvested and UHPLC‐HRMS‐based metabolite analyses performed to track the metabolic fate of the MeJA (Fig. 6b). MeJA treatment led to an accumulation of JA and 12‐OH‐JA in the flowers of *aos gh3.10 jar1* plants, indicating that MeJA was converted to free JA and further hydroxylated. Despite the abundance of JA, neither JA‐Ile nor 12‐OH‐JA‐Ile were detected in the samples. However, the formation of JA‐Gln, JA‐Glu, and JA‐Asn could be observed, indicating that conjugation of JA to other amino acids and the generation of these potentially bioactive jasmonates still occurred in the absence of GH3.10 and JAR1. Subsequently, the presence of JA‐Asn, JA‐Glu, and JA‐Gln was analysed in leaves 2 hpw of *gh3.10 jar1* plants to check whether these conjugates are also formed without exogenous MeJA application. Here, the JA‐Gln was detected as a native jasmonate formed in leaves of *gh3.10 jar1* plants (Fig. S8). Its identity was further confirmed MS/MS experiments in wounded leaves and by synthesizing an authentic standard with recombinant GH3.15 (Fig. S9).

### External application of JA‐Ile and JA‐Gln to flowering plants does not restore fertility to *aos gh3.10 jar1* plants

**Fig. 7:**
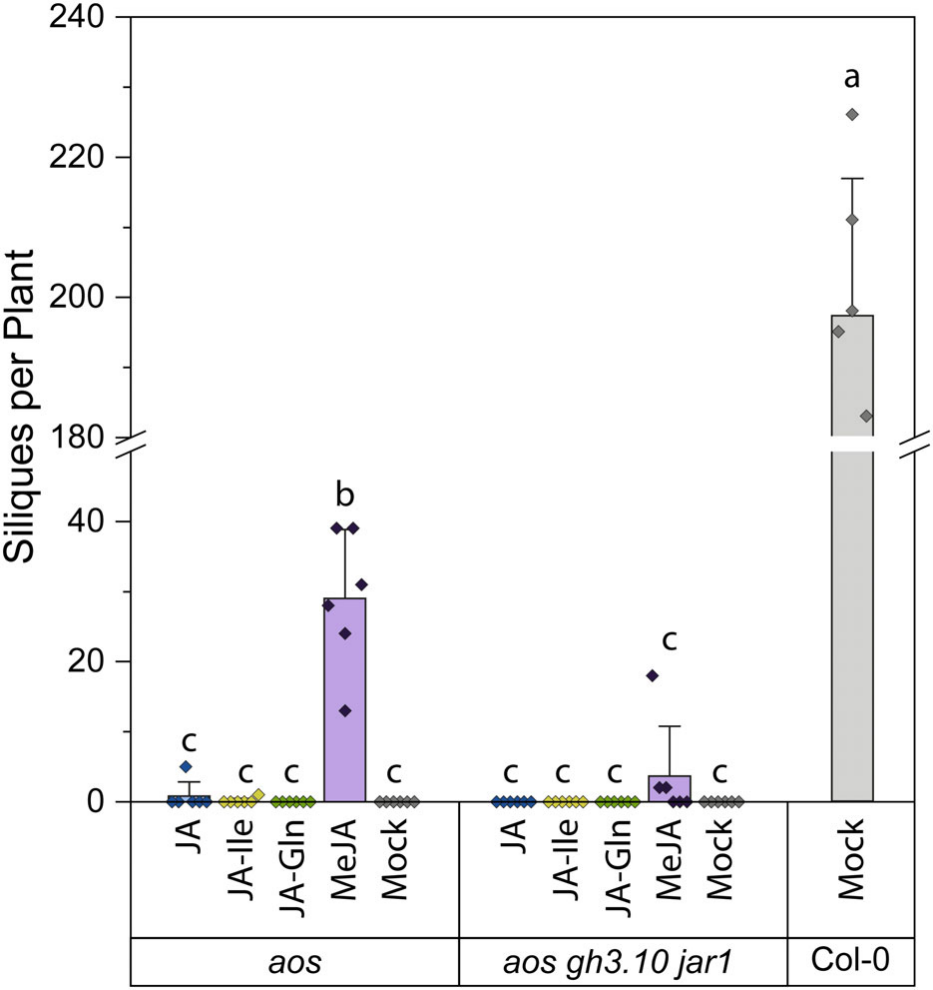
Silique formation of jasmonate‐treated *aos* and *aos gh3.10 jar1* lines. Six‐week‐old *aos gh3.10 jar1* plants were sprayed daily for 2 weeks with buffer containing 0.1% Tween‐20 and either 500 μM JA, 500 μM JA‐Ile, 500 μM JA‐Gln or 440 μM MeJA. Jasmonate‐free mock buffer treatment was included. The number of siliques per plant was subsequently determined. Mean ± SD of six plants are shown.

Since JA‐Gln was detected as the dominant signal of a jasmonoyl conjugate in flowers of *aos gh3.10 jar1* plants sprayed with MeJA, its potential function as a bioactive jasmonate was investigated. Recombinant GH3.15 and JAR1 were employed to produce JA‐Gln and JA‐Ile as positive control, because the formation of JA‐Gln by GH3.15 was previously reported (Sherp *et al*. 2018). In two initial experiments, *aos gh3.10 jar1* plants were either sprayed with or dipped in solutions containing either 1 mM JA‐Gln or JA‐Ile to check for silique formation. Neither JA‐Ile nor JA‐Gln application restored fertility. The experiment was subsequently repeated a third time and additional control treatments and genotypes were included. Six *aos* and *aos gh3.10 jar1* plants were sprayed daily over the course of 2 weeks with either 440 μM MeJA, 500 μM JA, JA‐Ile, JA‐Gln, or water containing 0.1% Tween‐20 as mock treatment. Mock‐treated Col‐0 plants were included as a wild‐type reference. At the end of the treatment, silique formation was determined and flowers were harvested for metabolite analysis. As previously observed, MeJA treatment restored fertility to all six *aos* plants (Park *et al*. 2002). This effect was diminished in *aos gh3.10 jar1* plants, and only three plants responded to the treatment (Fig. 7). Treatment with free JA restored fertility to only one out of six *aos* plants that produced eight siliques, suggesting a reduced uptake of the compound compared to MeJA. After JA‐Ile treatment, a single silique was formed on one *aos* plant, which was a unique occurrence out of three independent experiments and is therefore interpreted as a false‐positive. JA‐Gln treatment did not restore fertility. Taken together, only MeJA treatment was able to restore fertility in *aos gh3.10 jar1* plants, while neither treatment with the active COI1‐ligand JA‐Ile nor with JA and JA‐Gln showed the expected effect.

### JA, JA‐Ile, and JA‐Gln are taken up and metabolized by flowers without restoring fertility in *aos gh3.10 jar1* plants

**Fig. 8:**
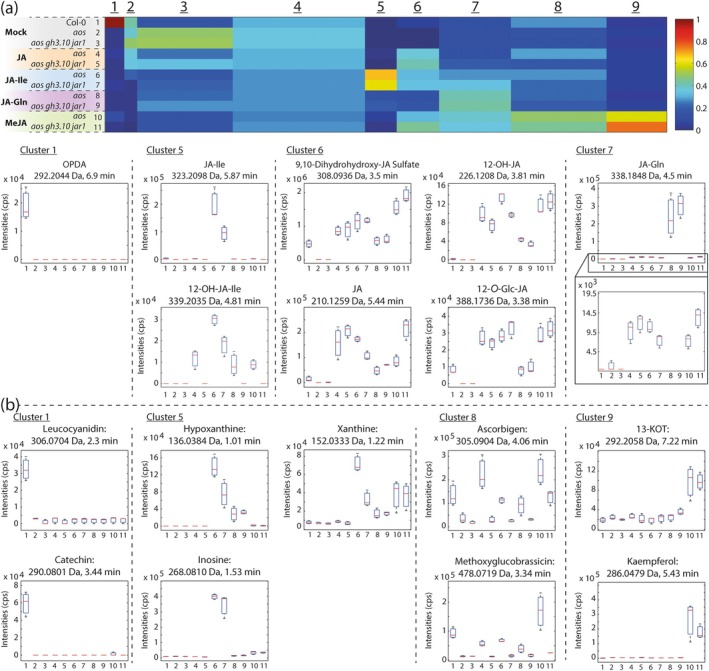
Non‐targeted metabolomics confirmed uptake of jasmonates into flower tissues upon treatments. Col‐0, *aos* or *aos gh3.10 jar1* plants were sprayed with buffer containing 440 μM MeJA, 500 μM JA, JA‐Ile or JA‐Gln, and 0.1% Tween‐20 daily for 2 weeks. Jasmonate‐free mock buffer treatment was included. Flowers of stage 13–15 were harvested 2 h after the last treatment. Samples of two plants per treamment were pooled. Metabolites were analysed by a non‐targeted metabolite fingerprinting approach based on UHPLC‐HRMS. 782 metabolite features (FDR < 10^−5^) were clustered in nine clusters by means of one‐dimensional self‐organizing maps (a). Average intensity values are indicated by the colour scale. The width of each cluster is proportional to the number of features assigned to this cluster. Box plots for selected metabolites of the indicated clusters are shown. Borders represent the high and low value of the measurement, and horizontal lines represent the median value. Selected intermediates of jasmonate catabolism and further metabolites (b) are shown by cluster. The identity of the metabolites was confirmed by MSMS fragmentation analysis. Data represent three biological replicates (pooled samples from two plants per replicate) and are shown in Dataset S1.

In three independent experiments with different application strategies, JA‐Gln and JA‐Ile did not restore fertility to *aos gh3.10 jar1* plants. To check the fate of the applied compounds, we performed non‐targeted metabolite fingerprinting with flowers of all treatment conditions, assuming that their metabolism could only be detected when the applied jasmonates were taken up into cells of this organ (Fig. 8). After UHPLC‐HRMS analysis in positive as well as negative ionization mode, a total of 782 metabolite features with a false discovery rate (FDR) < 10^−5^ were obtained. Features were clustered in nine clusters according to their intensity pattern by means of one‐dimensional self‐organizing maps representing the changes in the metabolite abundance in flowers of the different genotypes after jasmonate or mock treatments. Cluster 1 represents metabolites which accumulate exclusively in Col‐0. For instance, OPDA, a metabolite downstream of AOS, was only detected in Col‐0 flowers since it cannot be formed in the *aos* background. The same was true for the flavonoids leucocyanidin and catechin. Clusters 2 to 4 showed broader, less treatment‐specific accumulation of metabolite features. Cluster 5 represented predominantly JA‐Ile treatment‐dependent compounds. JA‐Ile itself showed the highest relative signal intensity, but was also detected in JA‐, JA‐Gln‐, and MeJA‐treated *aos* flowers at levels similar to mock‐treated Col‐0 flowers (Fig. S10, Table S3). Notably, JA‐Ile remained absent in *aos gh3.10 jar1* flowers even after prolonged treatment with other jasmonates. Application of JA‐Ile as well as JA‐Gln, however, caused accumulation of a number of intermediates from guanine metabolism in *aos* and *aos gh3.10 jar1* flowers. Inosine accumulated specifically upon JA‐Ile treatment. In contrast, xanthine accumulated after treatment with JA‐Ile, JA‐Gln or MeJA. This indicates joint bioactivity of the compounds, either directly or downstream of their uptake. Especially in the case of hypoxanthine, its enrichment was detected specifically after JA‐Ile and JA‐Gln treatment and indicates a shared effect on the plant chemotype. This may indicate that both conjugates exhibit bioactivity by affecting guanine metabolism. All three metabolites are part of the adenine ribonucleotide degradation pathway (Fig. S11). Metabolite features with jasmonate treatment‐dependent accumulation patterns, such as free JA, which was found after JA, JA‐Gln, JA‐Ile, and MeJA treatment, as well as endogeneously in Col‐0, are represented by cluster 6. This strongly suggests uptake of both JA‐Ile and JA‐Gln. Furthermore, 12‐OH‐JA and its metabolites, 9,10‐dihydrohydroxy‐JA‐sulfate and 12‐*O*‐Glc‐JA, were detected after all jasmonate treatments (Strehmel *et al*. 2014; Wasternack & Feussner 2018; Haroth *et al*. 2019), as well as endogenously in Col‐0. Cluster 7 represented predominantly JA‐Gln treatment‐dependent metabolites. Hence, JA‐Gln was the main compound detected, however it also accumulated in all other jasmonate‐treated samples including *aos gh3.10 jar1*, indicating an endogenous GH3.10 and JAR1‐independent turnover of jasmonates to JA‐Gln. Cluster 8 contained metabolites with a genotype rather than treatment‐dependent accumulation: methoxyglucobrassicin, an indolic glucosinolate, and ascorbigen, a glucobrassicin catabolite, were detected in Col‐0 flowers, but significantly reduced in mock‐treated *aos* and *aos gh3.10 jar1* lines. Methoxyglucobrassicin accumulation was induced above wild‐type levels upon MeJA treatment in *aos* and increased to 50–70% of the wild‐type signal after JA, JA‐Ile and JA‐Gln spraying. Treatment with JA and MeJA induced accumulation of ascorbigen above wild‐type levels in *aos* flowers, and JA‐Ile and JA‐Gln treatment restored wild‐type levels of ascorbigen to *aos*. In contrast, *aos gh3.10 jar1* plants failed to accumulate higher amounts of ascorbigen after jasmonate treatment. This again suggested specific involvement of GH3.10 and JAR1 in the regulation of glucosinolate metabolism, which could be rescued by jasmonate application in the *aos* background. Lastly, cluster 9 showed metabolite patterns that were specifically MeJA‐induced. 13‐keto‐(9*Z*,11*E*,15*Z*)‐octadecatrienoic acid (13‐KOT) and kaempferol accumulated after MeJA treatment. In both cases; MeJA spraying significantly increased metabolite accumulation above wildt‐ype levels. 13‐KOT is a keto‐polyunsaturated fatty acid that is derived from the activity of lipoxygenases (LOX), and its formation can be induced by jasmonates (Wasternack & Feussner 2018). Taken together, the presence of the above‐described jasmonates and jasmonate‐inducible metabolite accumulation suggest that all applied jasmonates were at least partially taken up by the plants, further metabolized, and served as signals. However, only MeJA treatment reliably restored fertility to *aos* and to a lesser extent to *aos gh3.10 jar1* plants. At this level, bioactivity of both JA‐Gln and JA‐Ile could not be assessed and therefore JA‐Asn and JA‐Glu were not further tested.

## DISCUSSION

The metabolism of jasmonates is characterized by remarkable plasticity that is shaped by both the synthesis and breakdown of these oxylipins (Wasternack & Feussner 2018). In addition to the major jasmonate‐forming enzyme JAR1, GH3.10 was recently identified as another contributor to JA‐Ile synthesis (Delfin *et al*. 2022). The loss of both enzymes, however, does not lead to the male‐sterile phenotype of jasmonate perception or biosynthesis mutants (McConn & Browse 1996). Indeed, Delfin *et al*. (2022) reported trace amounts of JA‐Ile in these plants. Although we cannot absolutely exclude residual amounts of JA‐Ile in the double mutant plants, the question is whether there is an additional signal molecule which induces silique formation in the absence of JA‐Ile. The isoleucine conjugate JA‐Ile is the principal COI1 ligand in angiosperms, but JA‐Leu, JA‐Val, and JA‐Ala were also shown to bind COI1 (Katsir *et al*. 2008; Saito *et al*. 2023). Comprehensive analyses of all possible interactions between COI1 and all JAZ proteins in the presence of JA‐Ile have been reported for rice and *Arabidopsis* (Wang *et al*. 2023). However, comparable information for other jasmonate conjugates is still lacking. Notably, our candidate for another bioactive jasmonate, JA‐Gln, has only been tested on COI1 with JAZ1 and JAZ3, where it did not facilitate interaction (Katsir *et al*. 2008). Moreover, in the liverwort *Marchantia polymorpha*, *dn*‐OPDA seems to be the COI1 ligand (Monte *et al*. 2018).

Transcriptional profiling of *gh3.10 jar1* versus wild‐type flowers revealed a strong decrease in transcripts related to jasmonate‐induced pathways, with over 40 jasmonate‐associated genes downregulated (Fig. 5, Fig. S10), but no other differential *GH3* or fertility‐related gene expression (Table S2). In addition, purified recombinant clade III GH3 enzymes, many of which are lacking functional annotation, did not show JA‐Ile‐forming activity *in vitro*. This is in contrast to a previous study that used *E. coli* lysates expressing these enzymes (Brunoni *et al*. 2023).

For a functional characterization of the physiological substrates of GH3.10, an alternative approach using non‐targeted *ex vivo* metabolomics was therefore pursued (Ni & Feussner 2023). Indeed, JA‐Ile was identified as the dominat product, detected across all four settings tested (flowers and leaves 2 hpw of both Col‐0 and *gh3.10 jar1*; Fig. 3). Intriguingly, further jasmonate conjugates, JA‐Met and JA‐Val, were identified as products of GH3.10 activity. Notably, 12‐OH‐JA was used up as substrate to form 12‐OH‐JA‐Ile, in addition to the previously reported CYP94‐dependent oxidation of JA‐Ile (Kitaoka *et al*. 2011; Koo *et al*. 2011; Widemann *et al*. 2015; Bruckhoff *et al*. 2016). 12‐OH‐JA‐Ile has gained recent attention as a bioactive jasmonate (Poudel *et al*. 2019; Saito *et al*. 2023), although its specific physiological function compared to JA‐Ile remains to be investigated (Jimenez‐Aleman *et al*. 2019; Saito *et al*. 2023). Furthermore, ddh‐JA‐Ile was identified as another direct product of JAR1 and GH3.10 in the *in vitro* assay. Previously, ddh‐JA was identified as an endogenous jasmonate formed in both Col‐0 and *opr3* plants upon wounding, although ddh‐JA‐Ile was not detected (Chini *et al*. 2018). Given that we detected ddh‐JA‐Ile through *in vitro* formation by JAR1 and GH3.10, it is possible that ddh‐JA‐Ile may act directly as another ligand of COI1, similar to 12‐OH‐JA‐Ile. This would provide an alternative explanation to the bioactivity observed for ddh‐JA and further expand the range of reported bioactive jasmonates (Chini *et al*. 2018). Recently OPDA‐Asp, −Glu, ‐Ile, ‐Phe, and ‐Val were described as additional oxylipins that accumulate in leaves upon wounding or, in the case of OPDA‐Ile in flowers (Floková *et al*. 2016; Mik *et al*. 2023). Interestingly, neither JAR1 nor GH3.10 used OPDA as substrate under our conditions, in agreement with previous reports (Staswick *et al*. 2002; Guranowski *et al*. 2007).

While JAR1 proved to be the major JA‐Ile producer, GH3.10 showed higher specific activity on 12‐OH‐JA (Fig. 4). This may suggest a distinct physiological function of GH3.10 over JAR1, with research regarding the function of 12‐OH‐JA‐Ile ongoing. In an attempt to shift substrate preference of GH3.10 towards JA, GH3.10 Ala^334^ was exchanged for a histidine, and Tyr^172^ was swapped for a valine inhabiting the position in JAR1. None of the mutations however shifted GH3.10 relative substrate preference from 12‐OH‐JA to JA, indicating that other residues play a role in substrate specificity. An explanation for missing the correct residues could be that docking of JA in *trans* conformation on GH3.10 might have led to selecting the ‘wrong’ mutations. However, we tried non‐guided *in silico* docking with 12‐OH‐JA‐Ile and GH3.10, but the substrate ended up in a completely different location. Therefore we decided to focus on the structure‐based model.

No other JA‐Ile forming activity was observed *in vitro* or *in planta*. The flower transcriptome of *gh3.10 jar1* plants strongly resembled that of jasmonate biosynthesis or signalling mutants. The expression of a number of defence‐ and jasmonate biosynthesis‐related genes was significantly downregulated (Fig. 5). However, fertility, a feature of jasmonate signalling in angiosperms, was not completely abolished, and the expression of jasmonate‐induced fertility genes such as MYB21 and MYB24 was not affected (Table S2). This strongly suggests an additional, JA‐Ile independent signal that may specifically regulate a fertility‐related gene expression. Nevertheless, the signal is oxylipin‐derived, since both jasmonate synthesis and perception mutants are male‐sterile. Residual jasmonate signalling in the absence of JA‐Ile may be explained by two alternative models that place the fertility‐promoting signal upstream or downstream of JA. It is possible that oxylipin precursors upstream of JA are able to partially activate the COI1 pathway in the absence of its principal ligand, JA‐Ile, similar to dn‐OPDA in *Marchantia*. Alternatively, additional jasmonoyl‐amido conjugates other than JA‐Ile that facilitate COI1‐JAZ interactions in *in vitro* pulldown assays and might also act as ligands of COI1 *in planta* (Katsir *et al*. 2008). To pinpoint the origin of the signal upstream or downstream of JA, the *aos gh3.10 jar1* triple mutant was generated. The *aos* component was necessary to remove all potentially bioactive, endogenous oxylipin precursors of JA, while *gh3.10 jar1* plants did not show detectable JA‐Ile accumulation in any of our experiments. The application of MeJA to *aos gh3.10 jar1* plants provided two key findings in this study. First, it partially restored fertility to plants, showing that residual fertility in *gh3.10 jar1* is JA‐derived. Second, it demonstrated that even at high MeJA concentrations plants failed to accumulate JA‐Ile, supporting the notion that JAR1 and GH3.10 are the only enzymes forming JA‐Ile in flowers. Most interestingly, analysis of MeJA‐treated *aos gh3.10 jar1* flowers revealed jasmonate conjugates JA‐Gln, JA‐Asn, and JA‐Glu (Fig. 6). JA‐Gln was selected for further testing as a candidate for bioactive jasmonate since it was the most abundant signal, its synthesis by GH3.15 was already described *in vitro*, and endogenous JA‐Gln was detected in wounded leaves of wild‐type and *gh3.10 jar1* plants (Fig. S1) (Sherp *et al*. 2018). Neither the treatment with JA‐Gln nor JA‐Ile restored fertility to *aos gh3.10 jar1* plants. In contrast to MeJA, neither conjugate is volatile and a comparably polar compound; hence, it was initially assumed that they were not taken up by the tissue. However, JA‐Ile treatment has successfully been applied before in *Arabidopsis* and tobacco, albeit at lower concentrations and not to flowers, to restore fertility (Wang *et al*. 2008; Vadassery *et al*. 2014; Guan *et al*. 2019; Jimenez‐Aleman *et al*. 2019). Metabolite profiling of JA‐, JA‐Ile‐, JA‐Gln‐ and MeJA‐treated flowers, however, strongly suggested uptake of all compounds, as we detected various catabolites which are derived from enzymatic turnover (Fig. 8). It is likely that the conjugates were taken up beyond the apoplastic space, since a number of jasmonates detected in flowers after jasmonate treatment are formed by enzymes with cellular localization. For example, CYP94 enzymes hydroxylating JA‐Ile as well as JA‐Ile hydrolyzing enzymes ILL6 and IAR3 are localized to the ER (Koo *et al*. 2014; Zhang *et al*. 2016). Despite the apparent uptake of JA, JA‐Ile and JA‐Gln, no treatment could rescue the *aos*‐derived sterility. JA‐Ile treatment facilitated the formation of a single silique on one *aos* plant among 12 plants tested, including six *aos gh3.10 jar1* plants. However, this finding was not reproducible. While JA treatment led to the formation of more siliques, this was again restricted to a single *aos* plant responding to the treatment. Only MeJA treatment facilitated silique development in *aos* and also, for *aos gh3.10 jar1*, flowers (Fig. S3). MeJA treatment was more effective in *aos* plants, with all six plants responding to the treatment. This demonstrated the importance of JA‐Ile for flower development and suggests that the remaining signal in *aos gh3.10 jar1* may require a longer build‐up period to work or that it is a less compatible ligand for COI1. Comparison of the effect of MeJA to that of free JA suggests either a role of the volatility of MeJA for the uptake or that its hydrophobicity intensifies its uptake through hydrophobic barriers of flower tissue, since MeJA‐treated plants produced siliques at much higher rates. MeJA might be taken up more effectively through stomata and/or spreads more effectively through the tissues (Wasternack & Hause 2002). While wounding of *Arabidopsis* shoots causes translocation of the jasmonate precursor OPDA to undamaged roots, JA‐Ile was not transported in a similar manner (Schulze *et al*. 2019). There are reports from solanaceous species, however, that found exogeneously applied JA‐Ile was a mobile signal in the wounding response of distal leaves (Sato *et al*. 2011). This suggests that while the bioactive form of JA may also be transported in *Arabidopsis*, it is also formed locally in its target tissue. Flower tissue is characterized by a very thick and hydrophobic coat that may hinder uptake of hydrophilic substances such as JA and JA‐Ile, in contrast to shoot and leaf tissue (Nawrath *et al*. 2013). Flower development is a tightly regulated process that requires spatially and temporally restricted contributions from the pantheon of phytohormones (Chandler 2011; Ryan *et al*. 2015). JA‐Ile is specifically involved in stamen development and pollen maturation (Mandaokar *et al*. 2006; Mandaokar & Browse 2009; Acosta & Przybyl 2019). While catabolites of JA‐Ile and JA‐Gln were detected after their respective treatments, it is possible that neither conjugate reached the tissues essential for flower development, thus falling short of their site of action. In a previous key study investigating the exact target tissues of jasmonate signalling during flower development, jasmonate perception in the anther epidermis was already sufficient for pollen viability and anther dehiscence (Jewell & Browse 2016). However, flowers exhibit a developmental stage‐specific response to exogenously applied jasmonate, with peak responsiveness before flower opening (Stintzi & Browse 2000; Browse 2009). Given the potentially reduced mobility of JA‐Ile and JA‐Gln in *Arabidopsis*, indeed both conjugates may have failed to reach the anther epidermis of the closed flowers during their jasmonate‐responsive stage. Taken together, based on fertility as a readout, the bioactivity of JA‐Gln could not conclusively be evaluated since neither application of the bioactive jasmonate JA‐Ile nor JA‐Gln restored fertility to both *aos* and *aos gh3.10 jar1* plants. However, the role of GH3.10 in the direct formation of 12‐OH‐JA‐Ile and the data collected from *aos gh3.10 jar1* plant lines highlight the tremendous plasticity of jasmonate metabolism and that the involvement of bioactive jasmonates in plant physiology is still an evolving field of research.

## AUTHOR CONTRIBUTIONS

BN, KF, EH, and IF. conceived and designed the experiments. BN, EH, MK, CH, KF, and BH performed the experiments. MH synthezised and provided the 4,5‐didehydro jasmonic acid standard used. BN, EH, MK, KF, MH, and IF analysed and discussed the data, BN, KF, and IF wrote the article. All authors edited and approved the manuscript.

## FUNDING INFORMATION

B.N. is supported by the International Max Planck Research School for Genome Science (IMPRS‐GS) in the framework of Goettingen Graduate School for Neurosciences, Biophysics, and Molecular Biosciences (GGNB) at the University of Göttingen. M.K. and B.H. were supported by the GGNB Program PRoTECT. I.F. acknowledges funding from the Deutsche Forschungsgemeinschaft (GRK 2172 PRoTECT; ZUK 45/2010; INST 186/822‐1; INST 186/1434‐1).

## CONFLICT OF INTEREST STATEMENT

The authors declare there are no competing interests.

## Supporting information


**Fig. S1.** Oxylipin accumulation in *aos*, *gh3.10 jar1* and Col‐0 plants up to 6 h post‐wounding. Mean ± SD of three technical replicates for each time point are shown. Relative levels of JA‐Gln and 12‐OH‐JA‐Ile were determined relative to the signal of the deuterated JA standard.
**Fig. S2.** JA‐Ile signal in phytohormone measurement samples including background from empty control and aos lines. Signals shown are representative for each of the triplicates measured for each genotype. Empty extraction control and aos negative control signals at the retention time of JA‐Ile were assessed to establish the signal background of every measurement. Only signals three‐fold above the background signal height were integrated and quantified.
**Fig. S3.** JA‐Val and JA‐Ile levels in flowers (upper panel) and leaves at 2 hpw (lower panel) of *gh3.10 jar1* plant lines. Col‐0 plants served as wild‐type control. Mean ± SD of three technical replicates are shown.
**Fig. S4.** JA‐Ile generated by GH3.10 in an *ex vivo* assay from leaves at 2 h post‐wounding (hpw) and flowers of Col‐0 (a) as well as gh3.10 jar1 plant material (b). (c) shows an overview of further jasmonoyl‐amino conjugates and their detection in the respective plant metabolite extracts.
**Fig. S5.** Molecular docking of the 12‐OH‐JA‐Ile conjugate in GH3.10 (a) and JAR1 (b) based on the available crystal structure of JAR1 (4EPL; Westfall *et al*. 2012). Asterisks in the structure of GH3.10 indicate the amino acids swapped for their respective counterparts from JAR1 as indicated in the alignment (c).
**Fig. S6.** *In vitro* characterization of GH3.10 and JAR1 jasmonate substrate preference. The influence of GH3.10 active site residues on the conversion rate of 12‐OH‐JA and JA was tested. 100 μg purified recombinant JAR1, GH3.10, GH3.10 A334H, GH3.10 Y172V, and GH3.10 A334H Y172V were incubated in reaction buffer containing 0.1 mM Ile and 1 mM JA or 12‐OH‐JA. Reactions were incubated for 1 h at 30°C while shaking gently and stopped by addition of acetonitrile. Product formation was measured by UHPLC‐HRMS, and signal area relative to respective mean of the GH3.10 signal for each substrate is shown. Mean values ± SD three technical replicates are presented. Letters indicate significant differences at p > 0.05 as determined by one‐way ANOVA with Tukey's post‐hoc test.
**Fig. S7.** Schematic representation of jasmonate biosynthesis and activation of jasmonic acid by GH3 enzymes JAR1 and GH3.10. The obstructions in jasmonate biosynthesis introduced in the *aos gh3.10 jar1* triple mutant are indicated by red lines.
**Fig. S8.** Accumulation of oxylipins in Col‐0, *gh3.10 jar1* and *aos* plants at 2 h post‐wounding (hpw). While in the gh3.10 jar1 mutation leaves the accumulation of JA‐precursors OPDA and dnOPDA are unaffected, notably JA‐Ile can no longer be detected, whereas JA‐Gln is still present.
**Fig. S9.** JA‐Gln formed in leaves of Col‐0 at 2 h post‐wounding (a, c) or by recombinant GH3.15 in an *in vitro* incubation (b, d). (a, b) show EICs of the chromatograms and (c, d) the corresponding fragment spectra.
**Fig. S10.** Endogenous amounts of JA‐Ile in flowers after mock, JA, JA‐Gln, or MeJA treatment. Zoomed figure of JA‐Ile metabolite pattern shown in Fig. 8. Col‐0, *aos* or *aos gh3.10 jar1* plants were sprayed with buffer containing 440 μM MeJA, 500 μM JA, JA‐Ile, or JA‐Gln and 0.1% Tween‐20 daily for 2 weeks. Jasmonate‐free mock buffer treatment was included. Flowers of stage 13–15 were harvested 2 h after the final treatment. Samples of two plants per treatment were pooled. Metabolites were analysed by a non‐targeted metabolite fingerprinting approach based on UHPLC‐HRMS. Box plots for *aos* and *aos gh3.10 jar1* treated with JA‐Ile are out of range (indicated by arrows) and not shown. Borders represent high and low values of the measurement and horizontal lines represent the median value. Selected metabolites with distinct treatment responses are shown. The identity of the metabolites was confirmed by MSMS fragmentation analysis. Data represent n = 3 biological replicates (pooled samples from two plants per replicate).
**Fig. S11.** Purine metabolism pathway highlighting intermediates found in *ex vivo* data. Downloaded from https://www.kegg.jp/pathway/map00230+C00020.
**Table S1.** Primer sequences and restriction sites used for pET28 expression plasmid cloning.
**Table S2.** Expression of fertility‐related genes in flowers of *gh3.10 jar1* plants compared to wild‐type (Col‐0). Differential expression of fertility‐related genes downstream of phytohormone pathways involved in Arabidopsis flower development were checked for differential expression that might contribute to residual fertility in *gh3.10 jar1* plants. None of these fertility‐related genes were significantly up‐ or downregulated compared to the wild‐type (log2 fold change ≥2, *p* < 0.01).
**Dataset S1.** Non‐targeted metabolomics of *Arabidopsis thaliana* flowers after spraying with Mock solution, JA, JA‐Ile, JA‐Gln, and MeJA daily for 2 weeks.

## Data Availability

The data that support the findings of this study are available in the Supporting Information of this article. Metabolite fingerprinting data can be found in the Metabolights database under the ID: MTBLS9749.
