# Supplementary material for: Arabidopsis GH3.10 conjugates jasmonates
Source: Plant Biol (Stuttg). 2025 Mar 17;27(4):476–91. doi: 10.1111/plb.70001 (PMC12096059; doi:10.1111/plb.70001)
Supplement: Supplementary file 1 — Fig. S1. Oxylipin accumulation in aos, gh3.10 jar1 and Col‐0 plants up to 6 h post‐wounding. Mean ± SD of three technical replicates for each time point are shown. Relative levels of JA‐Gln and 12‐OH‐JA‐Ile were determined relative to the signal of the deuterated JA standard. Fig. S2. JA‐Ile signal in phytohormone measurement samples including background from empty control and aos lines. Signals shown are representative for each of the triplicates measured for each genotype. Empty extraction control and aos negative control signals at the retention time of JA‐Ile were assessed to establish the signal background of every measurement. Only signals three‐fold above the background signal height were integrated and quantified. Fig. S3. JA‐Val and JA‐Ile levels in flowers (upper panel) and leaves at 2 hpw (lower panel) of gh3.10 jar1 plant lines. Col‐0 plants served as wild‐type control. Mean ± SD of three technical replicates are shown. Fig. S4. JA‐Ile generated by GH3.10 in an ex vivo assay from leaves at 2 h post‐wounding (hpw) and flowers of Col‐0 (a) as well as gh3.10 jar1 plant material (b). (c) shows an overview of further jasmonoyl‐amino conjugates and their detection in the respective plant metabolite extracts. Fig. S5. Molecular docking of the 12‐OH‐JA‐Ile conjugate in GH3.10 (a) and JAR1 (b) based on the available crystal structure of JAR1 (4EPL; Westfall et al. 2012). Asterisks in the structure of GH3.10 indicate the amino acids swapped for their respective counterparts from JAR1 as indicated in the alignment (c). Fig. S6. In vitro characterization of GH3.10 and JAR1 jasmonate substrate preference. The influence of GH3.10 active site residues on the conversion rate of 12‐OH‐JA and JA was tested. 100 μg purified recombinant JAR1, GH3.10, GH3.10 A334H, GH3.10 Y172V, and GH3.10 A334H Y172V were incubated in reaction buffer containing 0.1 mM Ile and 1 mM JA or 12‐OH‐JA. Reactions were incubated for 1 h at 30°C while shaking gently and stopped by addition of aceton [file PLB-27-476-s001.zip › PLB70001-sup-0004-Supporting Information Ni et al 2nd rev.pdf]

Supporting information

Arabidopsis GH3.10 conjugates jasmonates

Benedikt Ni[1], Moritz Klein[1], Ben Hossbach[1], Kirstin Feussner[1], Ellen Hornung[1], Cornelia Herrfurth[1,2], Mats Hamberg[3], and Ivo Feussner[1,2,4]

[1]Department of Plant Biochemistry, Albrecht-von-Haller-Institute, University of Goettingen, D-37077 Goettingen, Germany

[2]Service Unit for Metabolomics and Lipidomics, Goettingen Center for Molecular Biosciences (GZMB), University of Goettingen, D-37077 Goettingen, Germany

[3]Division of Physiological Chemistry II, Department of Medical Biochemistry and Biophysics, Karolinska Institutet, S-17177 Stockholm, Sweden

[4]Department of Plant Biochemistry, Goettingen Center for Molecular Biosciences (GZMB), University of Goettingen, D-37077 Goettingen, Germany

Correspondence: Ivo Feussner, Tel: +49-551-3925743, Email: ifeussn@uni-goettingen.de

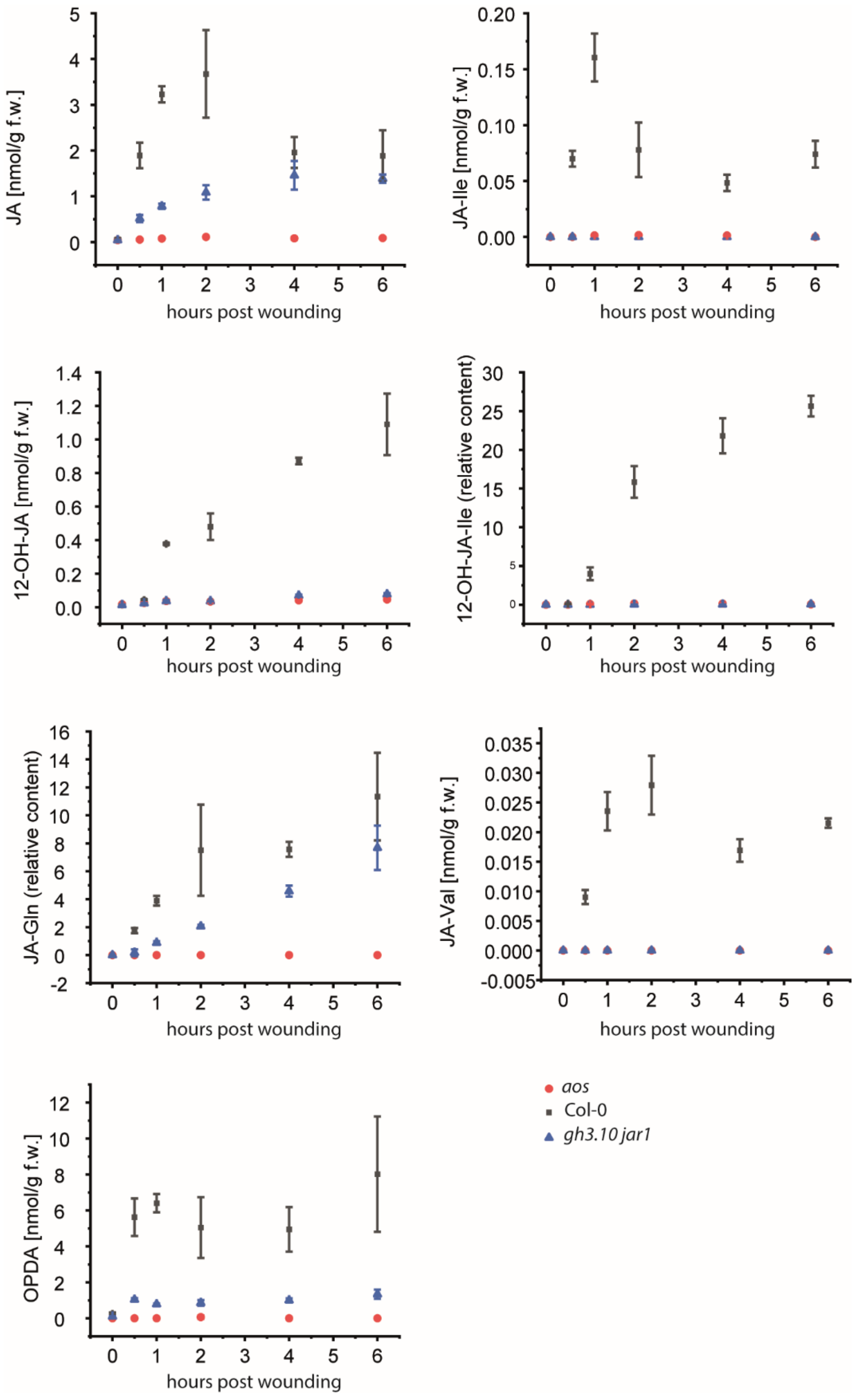

Fig. S1 Oxylipin accumulation in *aos*, *gh3.10 jar1* and Col-0 plants up to 6 h post wounding. Mean values and standard deviations of three technical replicates for each time point are shown. Relative levels of JA-Gln and 12-OH-JA-Ile were determined relative to the signal of the deuterated JA standard.

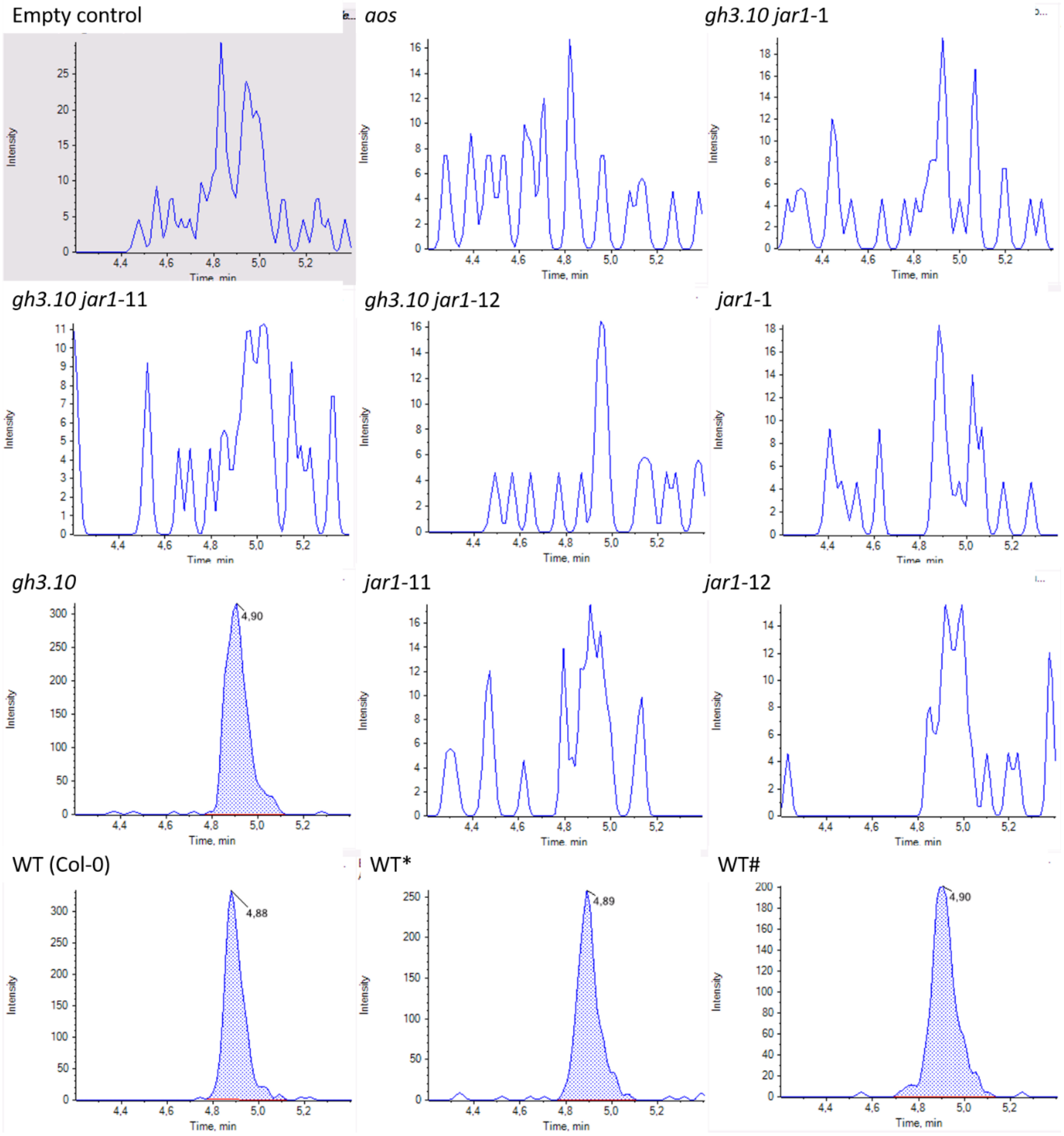

Fig. S2 JA-Ile signal in phytohormone measurement samples including background from empty control and aos lines. Signals shown are representative for each of the triplicates measured for each genotype. Empty extraction control and aos negative control signals at the retention time of JA-Ile were assessed to establish the signal background of every measurement. Only signals three times above the background signal height were integrated and quantified.

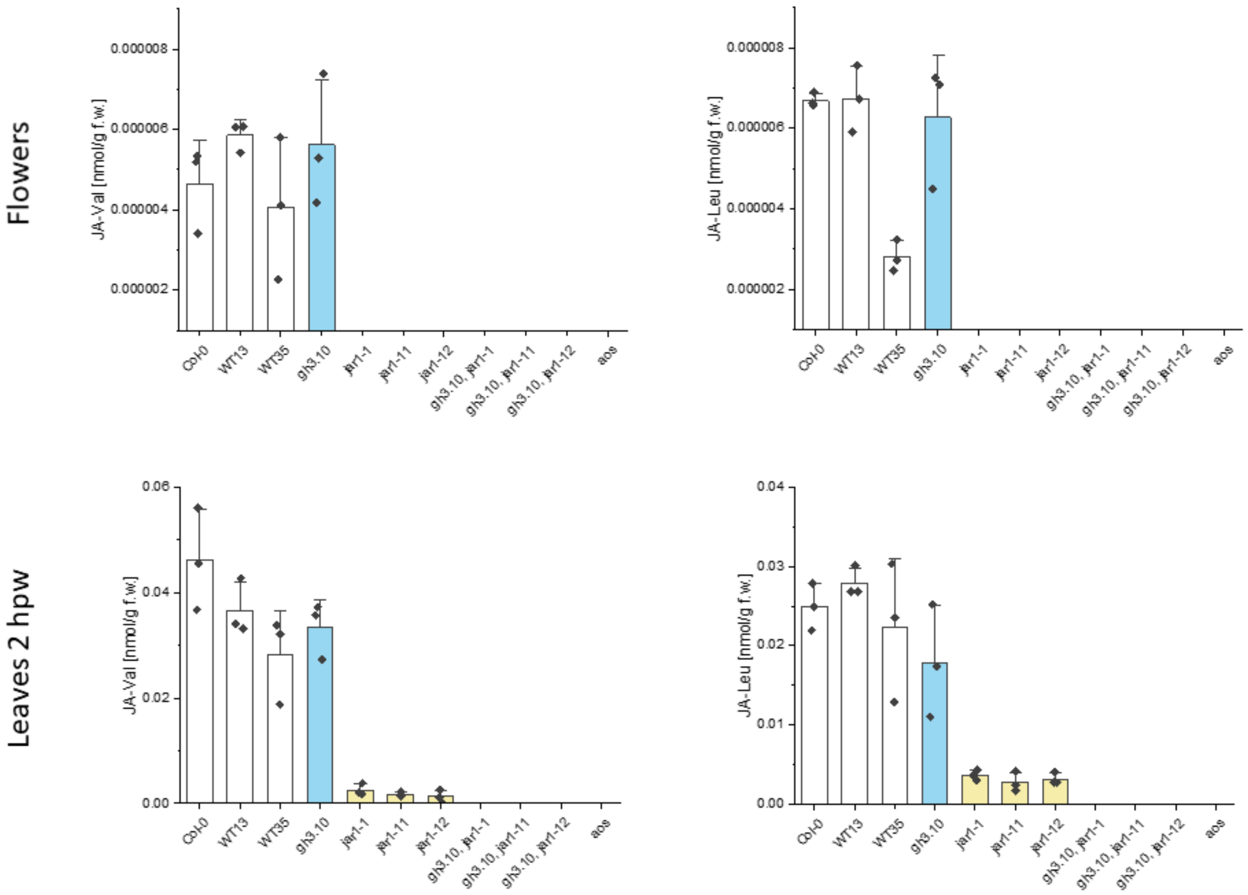

Fig. S3 JA-Val and JA-Leu levels in flowers (upper panel) and leaves 2 hpw (lower panel) of *gh3.10 jar1* plant lines. Col-0 plants served as wildtype control. Mean values and standard deviations of three technical replicates are shown.

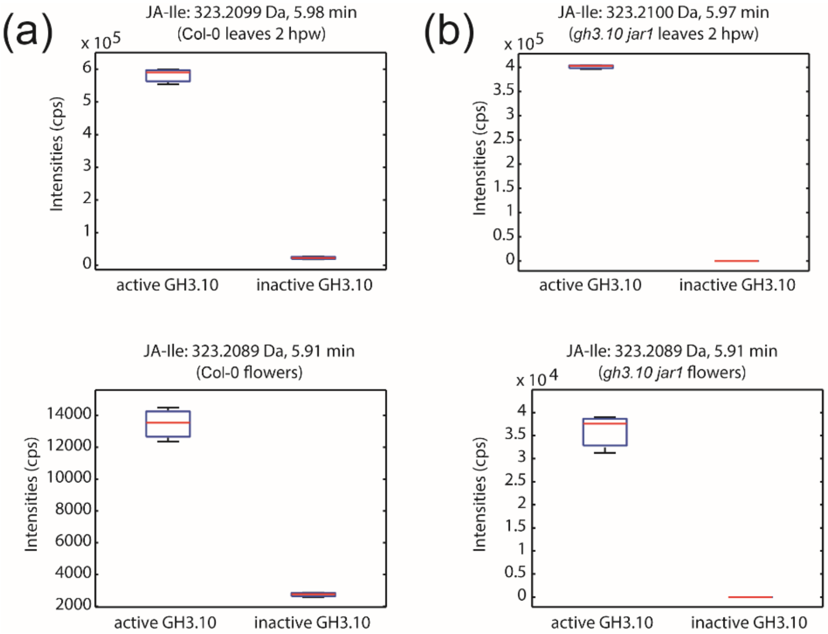

(c)

	gh3.10 jar1 flowers	*gh3.10 jar1* leaves 2 hpw	Col-0 flowers	Col-0 leaves 2 hpw
JA		✓		✓
JA-Val		✓		✓
JA-Ile	✓	✓	✓	✓
JA-Met		✓		✓
12-OH-JA				✓
12-OH-JA-Ile		✓		✓
12-OH-JA-Met		✓		✓

Fig. S4 JA-Ile generated by GH3.10 in an ex vivo assay from leaves 2 h post wounding (hpw) and flowers of Col-0 (a) as well as gh3.10 jar1 plant material (b). (c) shows an overview of further jasmonoyl-amino conjugates and their detection in the respective plant metabolite extracts.

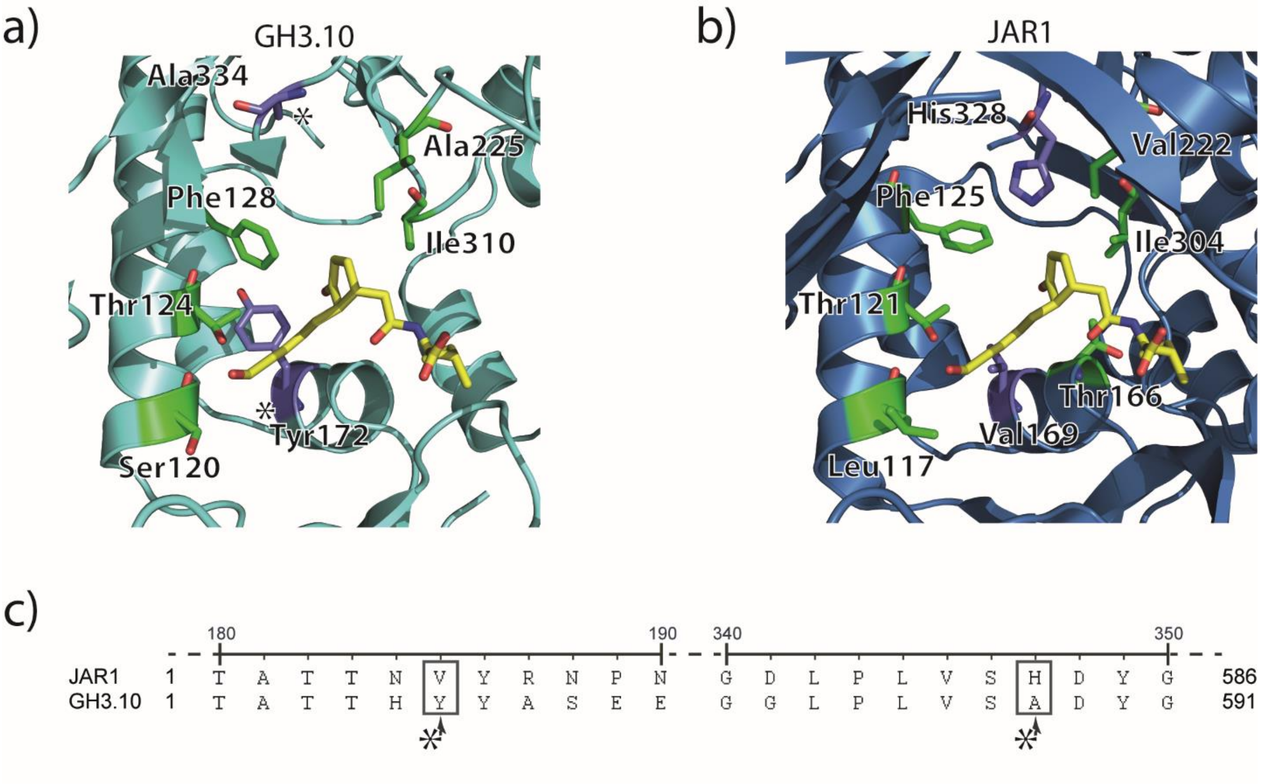

Fig. S5 Molecular docking of the 12-OH-JA-Ile conjugate in GH3.10 (a) and JAR1 (b) based on the available crystal structure of JAR1 (4EPL, (Westfall et al., 2012)). Asterisks in the structure of GH3.10 indicate the amino acids swapped for their respective counterparts from JAR1 as indicated in the alignment (c).

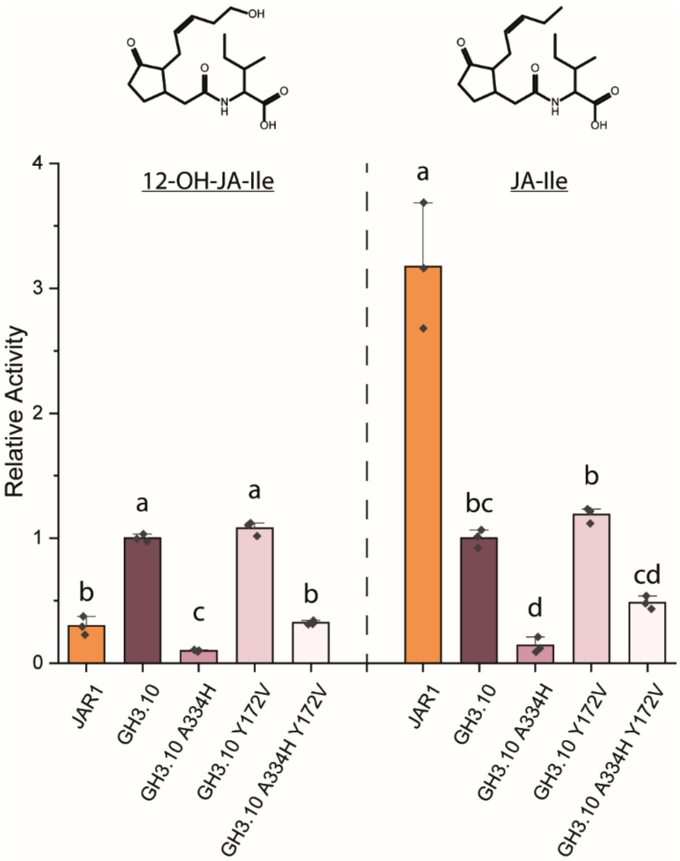

Fig. S6 *In vitro* characterization of GH3.10 and JAR1 jasmonate substrate preference. The influence of GH3.10 active site residues on the conversion rate of 12-OH-JA and JA was tested. 100 µg purified recombinant JAR1, GH3.10, GH3.10 A334H, GH3.10 Y172V and GH3.10 A334H Y172V were incubated in reaction buffer containing 0.1 mM Ile and 1 mM JA or 12-OH-JA, respectively. Reactions were incubated for 1 h at 30 °C while shaking slightly and stopped by addition of acetonitrile. Product formation was measured by UHPLC-HRMS and signal area relative to respective mean of the GH3.10 signal for each substrate is shown. Mean values and standard deviations of three technical replicates are presented. Letters indicate significant differences at $p > 0.05$ as determined by one-way ANOVA with Tukey's post-hoc test.

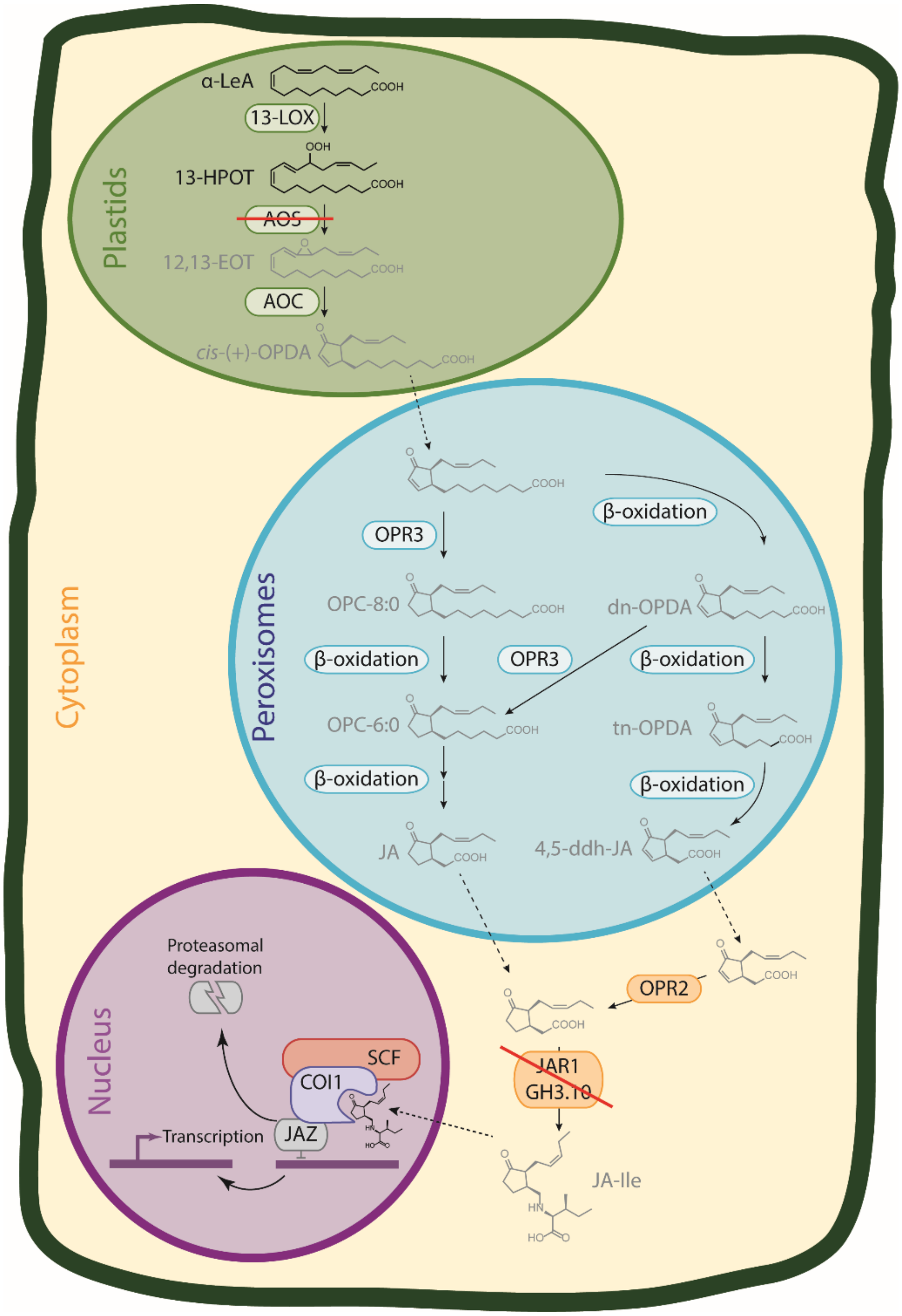

Fig. S7 Schematic representation of jasmonate biosynthesis and activation of jasmonic acid by GH3 enzymes JAR1 and GH3.10. The obstructions in jasmonate biosynthesis introduced in the *aos gh3.10 jar1* triple mutant are indicated by red lines.

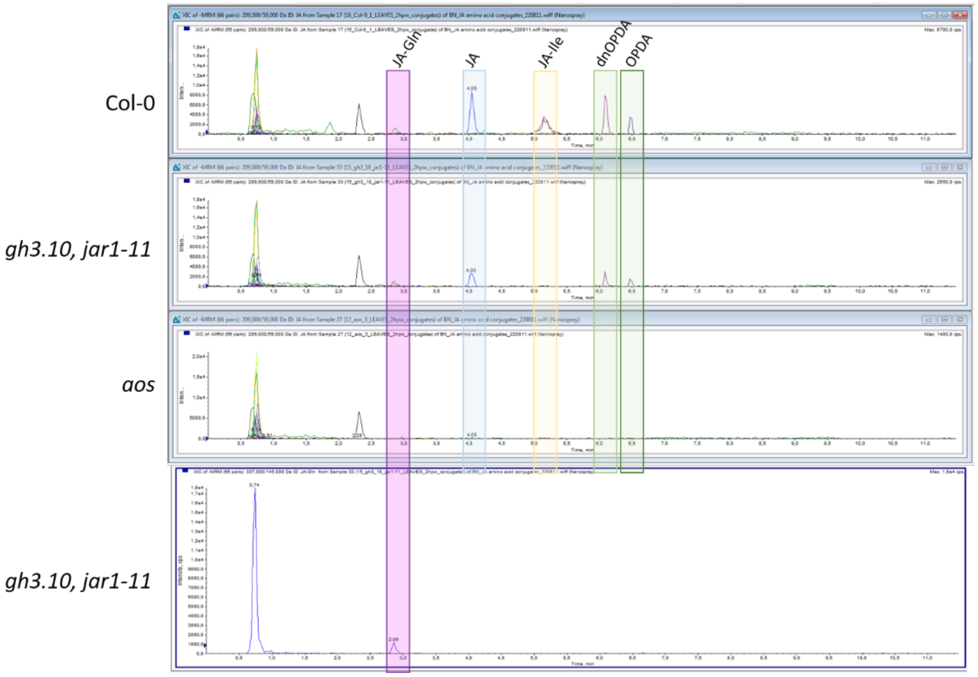

Fig. S8 Accumulation of oxylipins in Col-0, *gh3.10 jar1* and *aos* plants 2 hours post wounding (hpw). While the gh3.10 jar1 mutation leaves the accumulation of JA-precursors OPDA and dnOPDA unaffected, notably JA-Ile can no longer be detected, whereas JA-Gln is still present.

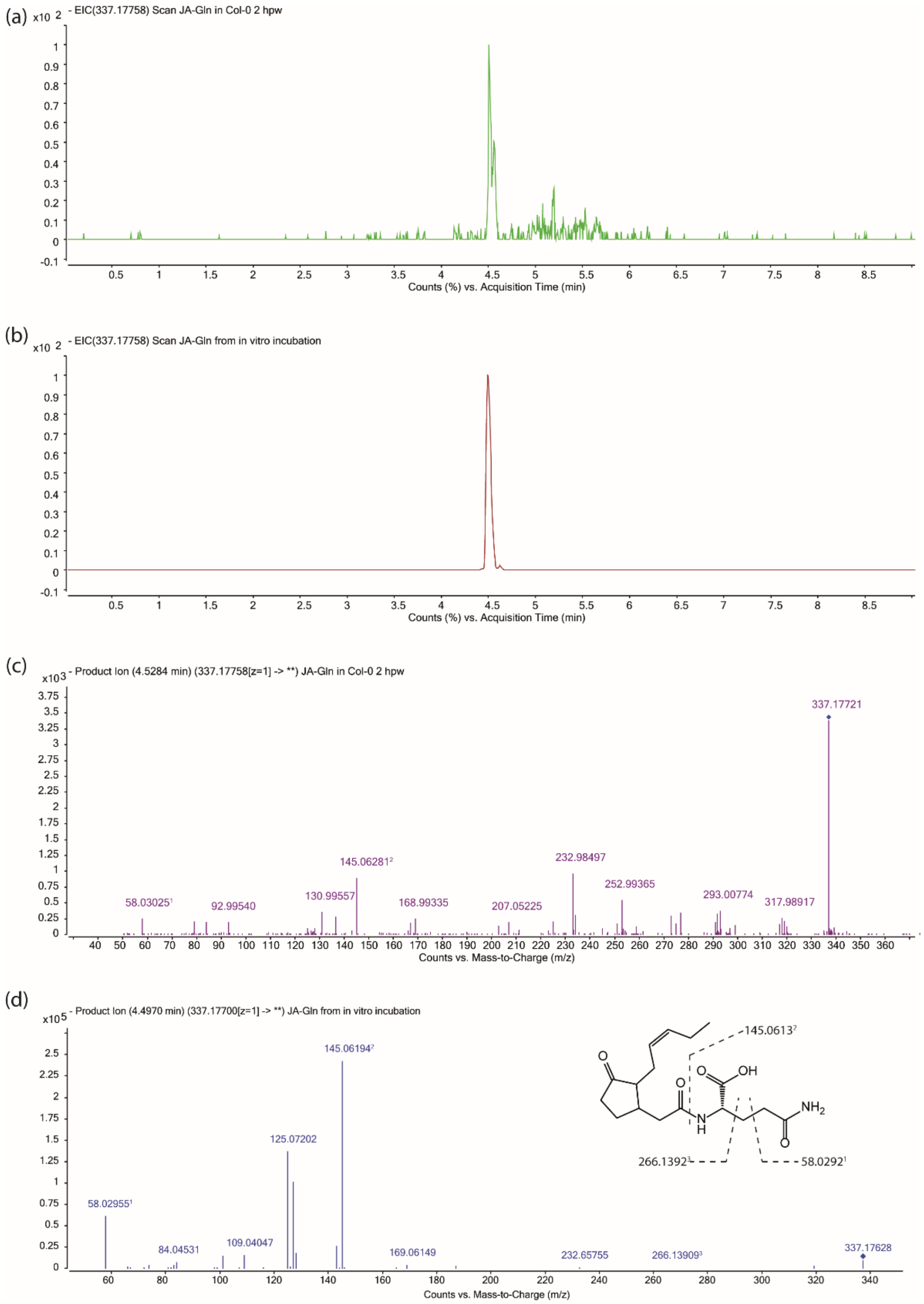

Fig. S9 JA-Gln formed in leaves of Col-0 2 h post wounding (a, c) or by recombinant GH3.15 in an *in vitro* incubation (b, d). (a, b) show the EICs of the chromatograms and (c, d) the corresponding fragment spectra.

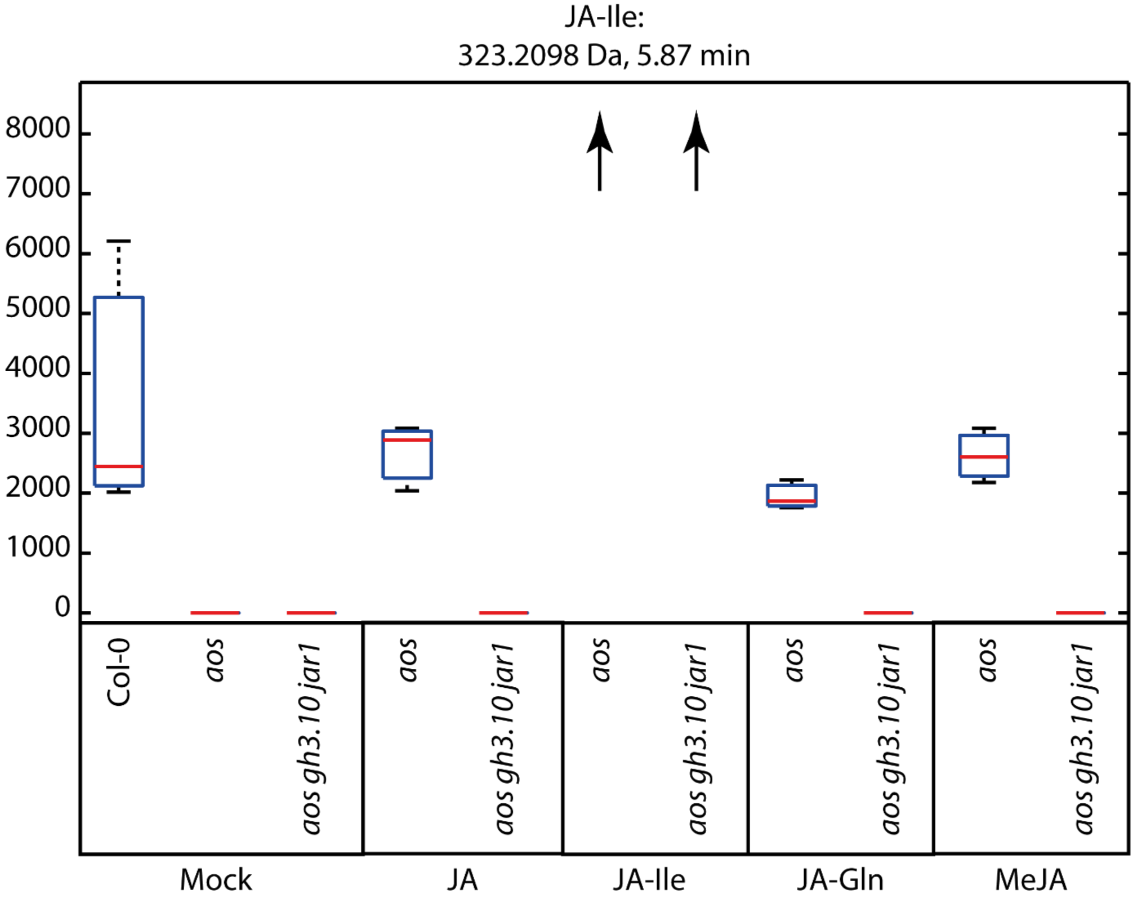

Fig. S10 Endogenous amounts of JA-Ile in flowers after mock, JA, JA-Gln or MeJA treatment. Zoomed in figure of JA-Ile metabolite pattern shown in Fig. 8. Col-0, *aos* or *aos gh3.10 jar1* plants were sprayed with buffer containing 440 µM MeJA, 500 µM JA, JA-Ile or JA-Gln and 0.1 % Tween-20 daily for two weeks. Jasmonate-free mock buffer treatment was included. Flowers of stage 13 - 15 were harvested 2 h after the last treatment. Samples of two plants per treatment were pooled. Metabolites were analyzed by a non-targeted metabolite fingerprinting approach based on ultra-high performance liquid chromatography high resolution mass spectrometry (UHPLC-HRMS). Box plots for *aos* and *aos gh3.10 jar1* treated with JA-Ile are out of range (indicated by arrows) and not shown. Borders represent the high and low value of the measurement and horizontal lines represent the median value. Selected metabolites with distinct treatment responses are shown. The identity of the metabolites was confirmed by MSMS fragmentation analysis. Data represent n = 3 biological replicates (pooled samples from two plants per replicate).

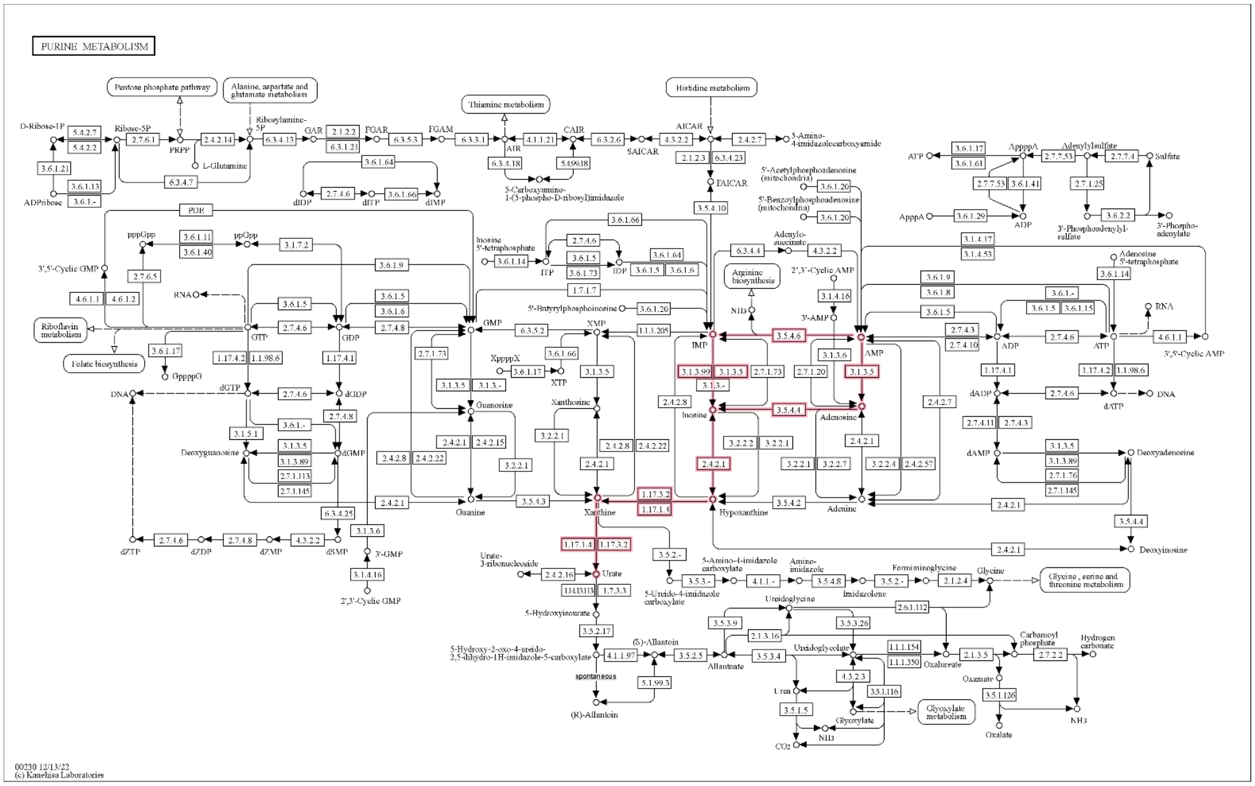

Fig. S11 Purine metabolism pathway highlighting intermediates found in *ex vivo* data. Downloaded from https://www.kegg.jp/pathway/map00230+C00020.

Table S1 Primer sequences and restriction sites used for pET28 expression plasmid cloning.

AtGH3	FORWARD	RESTRICTION	REVERSE	RESTRICTION
GH3.8	5'-*CATATG*ATGAGTTTATGCTCTGATCTCACTG-3'	NdeI	5'-*GTCGAC*TTATGACGAAAAGTTCAAAGAAAGGG-3'	SalI
GH3.13	5'-*CATATG*ATGTTGCCAAAGTTTGATCTAACAG-3'	NdeI	5'-*GTCGAC*TTATCCCCACGGCGGAGTTTCC-3'	SalI
GH3.14	5'-*CATATG*ATGTTTTTATCTCCTCGTAGTGCTC-3'	NdeI	5'-*GTCGAC*TTATCGGCTGGAATGCAGCTCATG-3'	SalI
GH3.16	5'-*GCTAGC*ATGACACCTTTTATCTGCACAGAG-3'	NheI	5'-*AAGCTT*TTAACGGCTGGAATGTAGCTCATG-3'	HindIII
GH3.17	5'-*CATATG*ATACCAAGTTACGACCCAAATG-3'	NdeI	5'-*GAATTC*CTAAGAATCTAAACCAAGTGGTTCC-3'	EcoRI
GH3.18	5'-*GCTAGC*ATGATGAATCCAAGTTTAAATCTGATGG-3'	NheI	5'-*AAGCTT*CTATGAGTCAAGAGGAGGAGATTTTTC-3'	HindIII
GH3.19	5'-*CATATG*ATGAGTTTAAGCGTTGAGCTCAAGG-3'	NdeI	5'-*GTCGAC*CTACAGCCAAAAATCTCCTCCTCTTAACTTA-3'	SalI

Table S2: Expression of fertility-related genes in flowers of *gh3.10 jar1* plants compared to wildtype (Col-0). Differential expression of fertility-related genes downstream of phytohormone pathways involved in Arabidopsis flower development were checked for differential expression that might contribute to residual fertility in *gh3.10 jar1* plants. None of these fertility-related genes were significantly up- or downregulated compared to wildtype (log2 fold change ≥ 2, $p < 0.01$) .

gene_id	log2FoldChange	padj	gene_name
AT1G30330	0.483156444	0.000416422	ARF6
AT5G37020	0.322249857	0.024362503	ARF8
AT3G27810	-0.187155804	0.56717099	MYB21
AT5G40350	-0.32107689	0.641735205	MYB24
AT2G16910	-0.856031824	0.10515846	AMS
AT1G63910	0.091136088	0.804395698	AtMYB103
AT4G28395	-0.707295165	0.115450307	ATA7
AT3G42960	-1.172297724	0.099754832	TA1
AT3G51590	-0.56736878	0.208915299	LTP12
AT1G07340	-1.217063893	0.235624074	STP2
AT3G23770	-0.680307615	0.458413189	-
AT2G18550	1.413659382	0.133814464	ATHB-21
AT5G04560	0.545864736	0.000185949	DME
AT2G36490	0.464673972	0.005014021	ROS1
AT5G61430	-0.128274229	0.734963373	NAC100
AT1G61110	-0.820638362	0.045164556	NAC025
AT2G25900	-0.018403606	0.972517745	ATCTH
AT5G62320	-0.98109399	0.097491983	MYB99
AT2G16030	-0.551918353	0.311888698	PLOU
AT2G07040	-0.108380359	0.722831885	PRK2
AT2G18040	-0.29307526	0.211878273	PIN1
AT2G34650	0.034244688	0.906307324	PID
AT4G31820	0.050402939	0.874302096	NPY1
AT4G37590	0.103121852	0.323250786	NPY5
AT5G11320	0.820512298	0.040568742	YUC4
AT5G25620	-0.187367984	0.465442543	YUC6
AT4G13260	-0.538934236	0.27370786	YUC2
AT4G32540	-1.100982442	0.096923762	YUC
AT5G03680	0.149117469	0.409817267	PTL
AT2G01570	0.176763885	0.068666806	RGA
AT4G02780	-0.384088509	0.326586174	GA1
AT1G44090	-1.524850854	0.295246637	GA20OX5
AT1G60980	-1.062883991	0.590300404	GA20OX4
AT5G07200	-0.735991724	0.727625979	GA20OX3
AT5G51810	-0.154166936	0.737564867	GA20OX2
AT5G21482	0.044633434	0.839169561	CKX7
AT3G63440	-0.007068338	0.995980411	CKX6

AT2G19500	-0.255442927	0.05370296	CKX2
AT4G29740	-0.783237795	0.077939113	CKX4
AT1G32210	-0.255616672	0.265624208	DAD1
